# Design, Implementation and Calibration of Analog Gyro-Based Angular Rate Data Acquisition System for High-Spinning Rotation Rate Applications

**DOI:** 10.3390/s26165083

**Published:** 2026-08-11

**Authors:** Ahmed Radi, Mostafa Mohamed, Shady Zahran

**Affiliations:** 1 Mobile Multi-Sensor Systems (MMSS) Research Group, Calgary, AB T2N 1N4, Canada; mostafa.mostafa@alumni.ucalgary.ca (M.M.); shady.zahran1@alumni.ucalgary.ca (S.Z.); 2 Technical Research Center, Cairo 11477, Egypt

**Keywords:** analog gyroscope, data acquisition, inertial measurement, rate measurement, sensor calibration, MEMS IMU

## Abstract

High-precision angular-rate measurements in extreme spin environments require systems capable of handling very high rotation rates, rapid startup, and reliable operation under vibration and shock. However, most commercially available gyro-based Inertial Measurement Units (IMUs) provide measurement ranges limited to approximately ±2000°/s, which may be insufficient for high-speed spinning platforms such as spin-stabilized satellites and drilling systems. This work presents the design, implementation, calibration, and validation of a complete Data Acquisition System (DAS) based on the ADXRS649 analog gyroscope, supporting angular rates up to ±20,000°/s. The system integrates a 12-bit ADC within a dsPIC33 microcontroller, high-speed nvSRAM for continuous logging, and firmware enabling sensor self-testing, memory checks, synchronized sampling, and onboard processing. Operating at a configurable sampling frequency of 50 Hz, the proposed system provides approximately 20 min of continuous data recording. Custom hardware, including multilayer PCB design, signal conditioning, power management, a rugged metallic enclosure, and polyurethane potting, enhances mechanical robustness for operation under vibration and shock. Laboratory calibration over the angular-rate range of ±980°/s quantified the gyroscope bias and scale factor, while experimental validation using a high-speed rotary machine demonstrated stable rolling measurements and reliable data integrity at angular rates exceeding 2000°/s. The results demonstrate that the proposed analog gyro-based DAS provides a robust and cost-effective solution for ultra-high-spin applications and future multi-sensor integration.

## 1. Introduction

Microelectromechanical Systems (MEMS) have revolutionized modern sensing technology by integrating mechanical sensing elements with electronic circuitry on a single chip, enabling compact, low-power, and cost-effective sensors for a wide range of industrial, biomedical, automotive, aerospace, and consumer applications [1]. Among the various MEMS devices, inertial sensors—particularly gyroscopes—have become indispensable for measuring angular motion in navigation, attitude determination, stabilization, and motion-tracking systems [2,3,4]. Continuous advances in MEMS fabrication technologies, structural designs [5], and signal-processing techniques have significantly improved the bandwidth, accuracy, robustness, and long-term reliability of MEMS gyroscopes, enabling their deployment in increasingly demanding operating environments [6,7,8,9].

Despite these advances, conventional MEMS gyroscopes and their associated embedded data acquisition hardware are primarily designed for moderate angular-rate applications. Most commercially available MEMS-based Inertial Measurement Units (IMUs) provide gyroscope measurement ranges of approximately ±250°/s to ±2000°/s, which are sufficient for conventional navigation and motion-control systems but become inadequate for extreme rotational environments. Once the applied angular rate exceeds the sensor’s full-scale range, output saturation occurs, resulting in clipped measurements and a significant degradation in angular-rate estimation accuracy [8,10].

Several engineering applications require substantially higher angular-rate measurement capabilities. Spin-stabilized projectiles, guided munitions, projectile telemetry systems, downhole drilling tools, and other rapidly rotating platforms may experience rotational rates ranging from several thousand to tens of thousands of degrees per second, depending on the operating conditions [11,12,13]. In addition to a wide measurement range, these applications demand rapid startup, continuous high-speed data acquisition, reliable non-volatile storage, and stable operation under severe vibration and shock. Consequently, the performance of the complete Data Acquisition System (DAS) becomes equally important as that of the sensing element itself.

To address these requirements, Analog Devices introduced the ADXRS649 analog MEMS gyroscope, which provides a measurement range of ±20,000°/s (extendable to ±50,000°/s), an ultrafast startup time of approximately 3 ms, continuous analog voltage output, and excellent resistance to vibration and shock [14]. However, fully exploiting the capabilities of such high-range analog gyroscopes requires a dedicated embedded data acquisition architecture capable of high-speed analog sampling, real-time processing, reliable non-volatile data storage, deterministic calibration, and rugged hardware integration suitable for harsh operating environments.

Although considerable research has focused on improving MEMS gyroscope structures and fabrication technologies [2,8,9,15], sensor calibration methodologies [6], and high-spin angular-rate estimation techniques [11,16,17], comparatively little attention has been devoted to the practical realization of complete embedded DAS specifically intended for ultra-high-spin applications. Such systems require the seamless integration of sensing hardware, analog signal conditioning, embedded processing, firmware, memory management, mechanical packaging, and experimental validation into a compact and reliable platform capable of operating under extreme rotational conditions.

Motivated by these requirements, this paper presents the design, implementation, calibration, and validation of a complete analog gyro-based DAS for high-spin applications. The proposed system integrates an ADXRS649 analog gyroscope (Analog Devices, Wilmington, MA, USA) with a dsPIC33 microcontroller incorporating a 12-bit Analog-to-Digital Converter (ADC) (Microchip Technology, Chandler, AZ, USA), high-speed nvSRAM (MikroElektronika, Belgrade, Serbia) based on the CY14B101Q 1-Mbit nvSRAM device (Infineon Technologies, Neubiberg, Germany) for continuous data logging, dedicated analog signal-conditioning circuitry, and embedded firmware supporting autonomous initialization, memory integrity verification, gyroscope self-test, synchronized sampling, and onboard data storage. A compact multilayer Printed Circuit Board (PCB) housed within a rugged metallic enclosure and encapsulated using polyurethane potting enhances mechanical robustness under vibration and shock. Deterministic calibration is performed using a precision laboratory turntable to estimate the gyroscope bias and scale factor, while system validation is conducted using a high-speed rotary machine operating beyond the dynamic range of conventional MEMS IMUs.

The remainder of this paper is organized as follows: Section 2 reviews the relevant literature on MEMS gyroscopes, high-spin angular-rate sensing, and related data acquisition systems. Section 3 presents the objectives of this work and summarizes its main contributions. Section 4 describes the proposed methodology, including the complete design of the angular-rate DAS, covering the hardware architecture, embedded firmware, PCB implementation, and the experimental procedures adopted for laboratory calibration. Section 5 presents the experimental evaluation, including the calibration results and validation of the proposed system under laboratory and high-spin operating conditions. Finally, Section 6 concludes the paper and outlines directions for future work.

## 2. Literature Review

Most commercially available MEMS-based IMUs are designed for consumer, automotive, robotics, and general industrial applications, where the maximum measurable angular rate typically ranges from ±250°/s to ±2000°/s. Representative examples include the TDK InvenSense MPU-6050 (up to ±2000°/s), TDK InvenSense ICM-42688-P (up to ±2000°/s), Bosch BMI088 (up to ±2000°/s), STMicroelectronics LSM6DSOX (up to ±2000°/s) and Analog Devices ADIS16470 (±2000°/s) (see Table 1). When the applied angular rate exceeds the configured full-scale range, the gyroscope output saturates at its maximum measurable value, preventing further tracking of the true angular motion. In other words, in the angular-rate range of more than ±2000°/s, conventional MEMS IMUs configured with a ±2000°/s full-scale measurement dynamic range experience overload (i.e., saturation), producing clipped measurements and significant estimation errors. This limitation is particularly critical for high-speed spinning platforms, such as spin-stabilized satellites and drilling systems, where reliable measurements beyond the conventional operating range are essential.

A closely related line of research focuses on integrated front-end circuits for high-performance MEMS and resonator gyroscopes. Ref. [17] presented a silicon MEMS disk resonator gyroscope with an integrated CMOS analog front end, achieving a compact, vacuum-sealed device with a high quality factor and good sensitivity suitable for precision inertial sensing (InvenSense, Inc., Sunnyvale, CA, USA). More recently, ref. [18] proposed a digital closed-loop sensing circuit for MEMS disk resonator gyroscopes, combining an integrated analog front end with digital control to enhance bias stability and noise performance. Similarly, ref. [19] demonstrated a 0.04°/h MEMS disk resonator gyroscope using an integrated CMOS analog front end to reach near navigation-grade stability. These studies collectively show the potential of highly integrated MEMS + front-end solutions to deliver excellent stability and noise characteristics; however, they are primarily optimized for bias stability and precision rather than for extremely large full-scale ranges or the specific constraints of high-spin, gun-launched, or similar applications. Recent advances in MEMS structural design have also focused on improving multi-axis sensing capability while maintaining compactness and high performance. For example, ref. [20] presented the design and fabrication of a single-drive tri-axis MEMS gyroscope, demonstrating that innovative structural configurations can simplify fabrication and improve sensing performance while reducing system complexity.

Recent surveys underline how central MEMS gyroscopes have become to modern inertial sensing while also emphasizing the persistent difficulty of maintaining accuracy under harsh and highly dynamic conditions. Ref. [7] review MEMS vibrating gyroscopes with a focus on performance metrics such as Angle Random Walk (ARW), Bias Instability (BI), and their degradation under temperature, vibration, and long-term operation, highlighting reliability as a key limiting factor in real-world deployments [2,21,22,23]. Huang et al. (2023) [2] broaden this perspective to MEMS and MOEMS gyroscopes, systematically cataloging device architectures and stressing that sensitivity, ARW, BI, dynamic measurement range, and bandwidth are the primary figures of merit for inertial navigation and guidance. Ref. [24] proposed a multi-signal repeated sampling framework based on the Generalized Method of Wavelet Moments (GMWM) to improve the estimation of stochastic error models, demonstrating enhanced identification of inertial sensor noise characteristics compared with conventional single-signal approaches. More recently, refs. [8,25] survey operating modes and structural innovations in MEMS gyroscopes, showing that advances in resonator design and processing technologies can achieve very low zero-bias instabilities and ARW in controlled conditions, but also noting that strong shock, large angular rates, and complex loading still pose significant challenges for maintaining performance. For MEMS-based gyro calibration and attitude estimation, ref. [26] proposed a calibration and attitude estimation framework for high-spin maneuvering bodies using a three-axis MEMS gyroscope. The method employs a two-stage Cascade Extended Kalman Filter (CEKF) to compensate for phase anomalies during free rotational motion, enabling simultaneous gyroscope scale-factor calibration and three-dimensional attitude estimation. The proposed approach was validated through turntable experiments at high-spin rates. Ref. [27] proposed a deep learning-based calibration method for MEMS IMU gyroscopes using a Temporal Convolutional Network (TCN) to model nonlinear and time-varying sensor errors. By learning error characteristics directly from historical gyroscope data, the proposed approach improves angular-rate compensation and enhances the accuracy of subsequent attitude and inertial navigation solutions. Together, these reviews make clear that while the intrinsic capability of MEMS gyroscopes continues to improve, robust operation in high-spin, high-shock environments remains an open problem.

On the signal-conditioning side, considerable effort has gone into interface and readout circuits that wrap MEMS gyroscopes in sophisticated analog and mixed-signal front ends. Ref. [28] propose a low-noise readout interface for a high-precision silicon MEMS vibratory gyroscope, integrating a closed-loop drive, sensing chain and high-resolution ADC to suppress front-end noise and enhance bias stability. Ref. [29] present a low-noise interface ASIC for a MEMS disk resonator gyroscope operating in force-to-rebalance mode, with self-excited drive, rate and quadrature loops plus an on-chip ΣΔ modulator and digital filter to digitize the rate signal, achieving very low rate-equivalent noise. In related work, ref. [30] design a MEMS gyroscope interface ASIC with analog closed-loop driving and on-chip temperature compensation, reporting bias instabilities on the order of a few degrees per hour and improved long-term stability. Ref. [31] further optimize the ΔΣ-ADC design inside MEMS gyro interface ASICs to support higher effective resolution and bandwidth for high-speed applications. Complementing these implementations, ref. [32] systematically analyze noise sources and suppression methods in MEMS gyro front-end circuits, reinforcing that achieving low noise and high stability depends as much on interface design as on the mechanical structure. These contributions collectively demonstrate that integrated MEMS + front-end solutions can deliver excellent noise and bias performance, but they are generally optimized for navigation-grade gyroscopes with moderate full-scale ranges (typically ±125–±400°/s) rather than for ultra-high-rate, gun-launched or high-spin platforms.

For extreme-dynamics scenarios, including high spin rates, several recent studies adopt high-range analog MEMS gyroscopes such as the ADXRS649 as the sensing element. Sun et al. [16] proposed a DAS based on the ADXRS649, using a high-resolution ADC and low-noise analog front end to reliably read gyro output and implement motion detection via interrupts, thereby reducing system power consumption. Their design demonstrates that an analog high-rate gyro such as the ADXRS649 can be interfaced effectively with digital electronics for inertial guidance tasks, but the work is largely confined to bench-level validation and does not address operation in extreme high-spin environments or long-duration autonomous logging. Ref. [11] address roll angular-rate measurement for high-spinning projectiles, where roll rates reach tens of thousands of degrees per second; they deploy a redundant cluster of three ADXRS649 gyroscopes and propose an optimization-based angular rate estimation method (OBARS) that combines ARIMA modeling with a Sage–Husa adaptive Kalman filter to mitigate noise and drift. Ref. [33] employ an ADXRS649, with ±20,000°/s range and 10,000 g shock survivability, in a parachute scanning measurement system—illustrating that such devices are well suited to short-duration, high-shock flight experiments. Ref. [34] integrate three ADXRS649 gyroscopes into a wireless “smart cricket ball” to measure spin rate throughout the ball’s trajectory under high angular accelerations. At the same time, signal-processing work by [35] uses turntable data acquired from an ADXRS649 to evaluate sparse-decomposition algorithms, explicitly modeling its output as a combination of null bias, random walk and power-supply noise.

A comparative summary of previously reported ADXRS649-based DAS is presented in Table 2. The comparison highlights that existing studies primarily focus on application-specific implementations and signal-processing techniques, with limited attention given to integrated stand-alone acquisition hardware, comprehensive power-on health verification, and experimental validation of the ADXRS649 sensor.

Industrial product documentation consistently describes the ADXRS649 family as “fast starting, ±20,000°/s vibration-rejecting rate gyros” with ultrafast startup on the order of 3 ms and powered shock survivability of 10,000 g, intended for harsh-environment motion sensing such as sports equipment, platform stabilization, and high-speed tachometry [14]. However, in every case, the gyroscope serves only as the sensing element, whereas the accompanying acquisition electronics are implemented using custom laboratory wiring, evaluation hardware, or application-specific electronics with limited architectural description. Furthermore, none of these studies presents a dedicated compact data-logging platform with integrated embedded firmware, autonomous non-volatile storage, or comprehensive system health monitoring with additional laboratory deterministic calibration.

Rather than ADXRS649, ref. [24] developed a hollow MEMS IMU for high-spinning projectiles employing an ADXRS645 gyroscope for roll-rate measurement and two ADXRS642 gyroscopes for pitch and yaw measurements. Combined with an adaptive Unscented Kalman Filter (AUKF), the proposed system was validated through simulation and semi-physical experiments for attitude estimation under high-dynamic conditions. Ref. [36] proposed a real-time attitude propagation algorithm for high-spinning flying bodies that combines MEMS gyroscopes with radial magnetometers to overcome gyroscope saturation during high-speed rotation, enabling accurate attitude estimation under extreme rotational dynamics.

Collectively, the existing literature reveals three clear research gaps. First, to the best of the authors’ knowledge, no previous study reports a compact, self-contained angular-rate data acquisition module that integrates sensing, embedded processing, power management, and high-speed non-volatile storage into a single ruggedized platform suitable for high-rate autonomous applications (up to ±20,000°/s). Second, although several studies describe signal-processing algorithms or interface electronics, none report firmware implementing complete power-on integrity verification of both the gyroscope and the non-volatile memory prior to mission execution, an important requirement for one-time-use and high-risk applications. Third, while previous studies primarily focus on sensing performance and signal-processing techniques, little attention has been given to the development of a ruggedized packaging solution capable of protecting the complete data acquisition system against severe vibration, mechanical shock, and temperature variations encountered in high-dynamic environments. The present work addresses these three gaps by developing a compact stand-alone angular-rate data acquisition kit integrating an ADXRS649 gyroscope, a dsPIC microcontroller with an embedded 12-bit ADC, and high-speed nvSRAM, together with power-on self-test routines for both the gyroscope and memory. The complete system is housed in a ruggedized metallic enclosure and encapsulated using a potting compound to enhance its resistance to vibration, shock, and temperature variations during operation. Finally, the proposed system is experimentally validated through laboratory calibration and real high-spin testing.

## 3. Objectives and Contributions

Building on the identified limitations of conventional MEMS gyroscopes in extreme high-spin environments, this study aims to design, implement, test, and calibrate a compact, stand-alone angular-rate data acquisition kit based on a high-measurement dynamic range analog MEMS gyroscope. Specifically, the work focuses on an ADXRS649-based system capable of measuring ultra-high rotation rates (up to ±20,000°/s), with ultrafast start-up, robust operation under strong vibration and shock, and reliable on-board data logging for subsequent trajectory and navigation analysis. The overarching objective is to demonstrate that a carefully engineered analog-gyro DAQ module—integrating sensing, embedded processing, self-test, and non-volatile storage—can provide field-ready, high-fidelity angular-rate measurements in demanding high-spin applications.

In line with this objective, the main contributions of this work can be summarized as follows:

**High-rate analog-gyro DAQ architecture**: We develop a compact hardware architecture that combines a wide-range ADXRS649 analog gyroscope, a dsPIC microcontroller with an integrated 12-bit ADC, and non-volatile nvSRAM. The design supports configurable sampling rates, real-time conversion of the analog gyro output, and high-throughput, non-volatile logging of angular-rate data suitable for short-duration, high-spin missions.

**Embedded self-test and health-monitoring framework**: We implement firmware routines that automatically verify the integrity of both the memory and the gyroscope before logging begins. This includes a memory read–write handshake and a gyroscope self-test procedure (using ST1/ST2 excitations and datasheet-based criteria), enabling the kit to autonomously confirm that the sensing and storage chain is functioning within acceptable limits.

**Systematic laboratory calibration methodology**: We propose and apply a calibration framework based on static and turntable-based dynamic tests, using multiple clockwise and counter-clockwise rotation scenarios. Bias and scale-factor parameters are estimated from repeated datasets, supported by a customized 3D-printed mounting fixture for the single-axis gyro card. This provides a practical, repeatable procedure for characterizing the error behavior of high-rate analog gyros in a controlled environment.

**Robust packaging and real in-field high-spin validation**: We design a metal enclosure and potting process (including pre- and post-potting tests) to protect the proposed gyro card and electronics under high shock and vibration, and we demonstrate the full kit in a real in-lab high-spinning test configuration. The recorded data confirm that the proposed system can operate reliably throughout the mission and deliver coherent angular-rate measurements that align with the expected motion profile.

Together, these contributions move beyond treating the ADXRS649 as a bare sensor element and instead present a complete, validated angular-rate data acquisition solution tailored to ultra-high-spin applications, bridging the gap between laboratory-grade MEMS research and field-deployable inertial instrumentation.

## 4. Methodology

This section describes the complete design of the proposed angular-rate DAS, from hardware architecture to embedded firmware and experimental procedures for calibration and validation. The emphasis throughout is on a self-contained, field-ready module that can be dropped into high-spin platforms with minimal external dependencies.

### 4.1. System Overview

The developed system is a single-axis “gyro card” designed to measure and log very high rotation rates using an analog MEMS gyroscope. The card integrates three key elements on a compact PCB:

- A high-range analog gyroscope (ADXRS649) providing the angular-rate signal;
- A 16-bit dsPIC microcontroller with an on-chip 12-bit ADC responsible for sampling, control, and basic health monitoring;
- A non-volatile nvSRAM (MikroElektronika, Belgrade, Serbia) based on the CY14B101Q 1-Mbit nvSRAM device (Infineon Technologies, Neubiberg, Germany) used as local high-throughput storage for angular-rate data.

The overall workflow is straightforward: after power-up, the microcontroller verifies the health of both memory and gyroscope, configures the acquisition parameters, and then enters a timer-driven interrupt routine in which the analog gyro output is sampled, digitized, and stored to nvSRAM at a fixed update rate.

### 4.2. Hardware Architecture

#### 4.2.1. Analog Gyroscope (ADXRS649)

**Figure 1:**
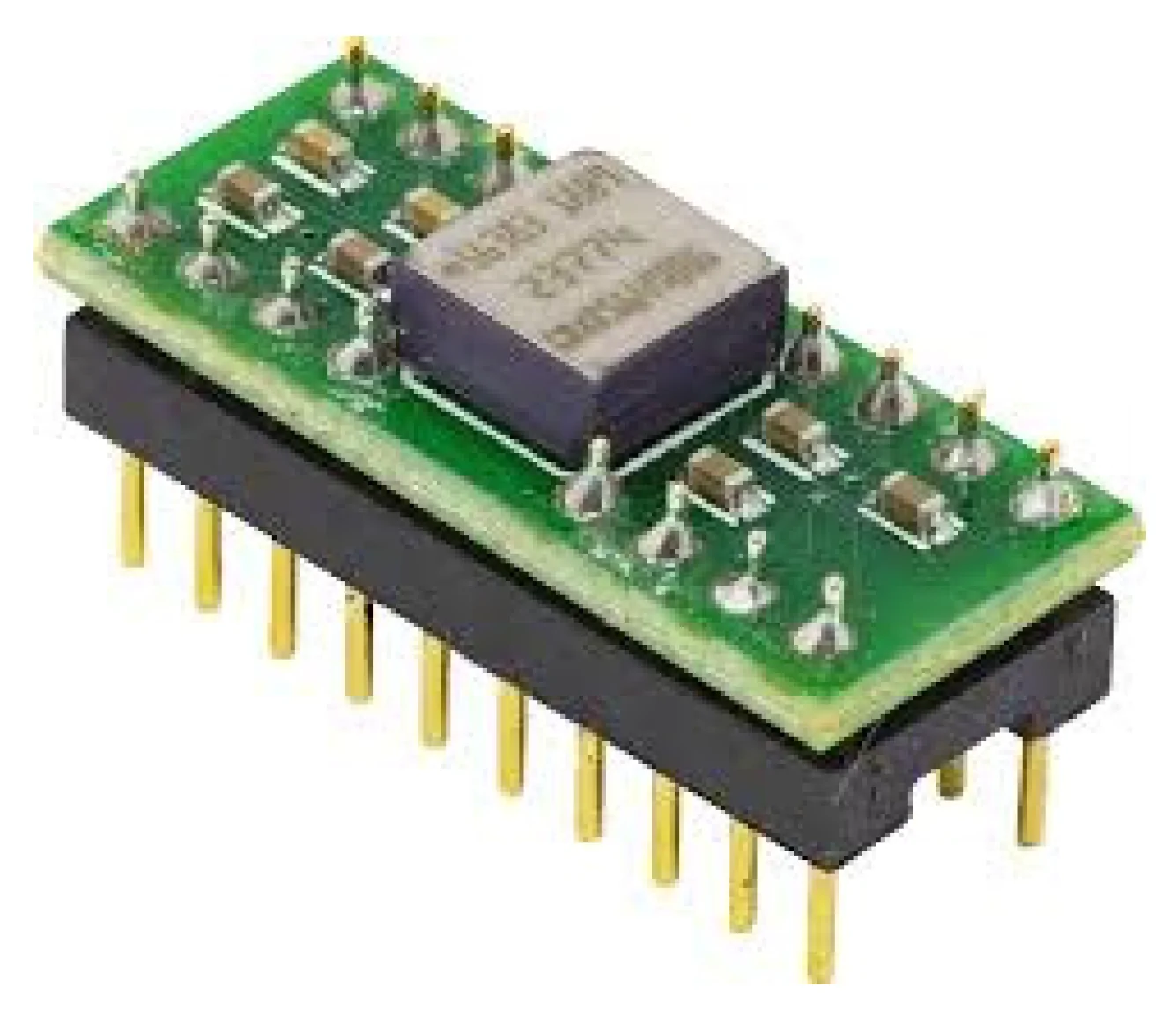
ADXRS649 single-axis analog angular-rate sensor.

The ADXRS649 is a single-axis, analog-output angular-rate sensor specifically designed for very high rotation rates and harsh environments (see Figure 1). It offers:

- A nominal measurement range of ±20,000°/s (with provisions to extend to ±50,000°/s);
- Ultrafast startup on the order of a few milliseconds;
- High shock survivability (10,000 g class) and robust vibration rejection.

The sensor is powered from a regulated 5 V rail, and its analog output is routed directly to the dedicated 12-bit ADC channel of the dsPIC. Careful PCB layout ensures short paths, low noise coupling, and appropriate decoupling around the gyro to preserve signal integrity under vibration.

#### 4.2.2. Microcontroller and ADC (dsPIC33EV256GM102)

**Figure 2:**
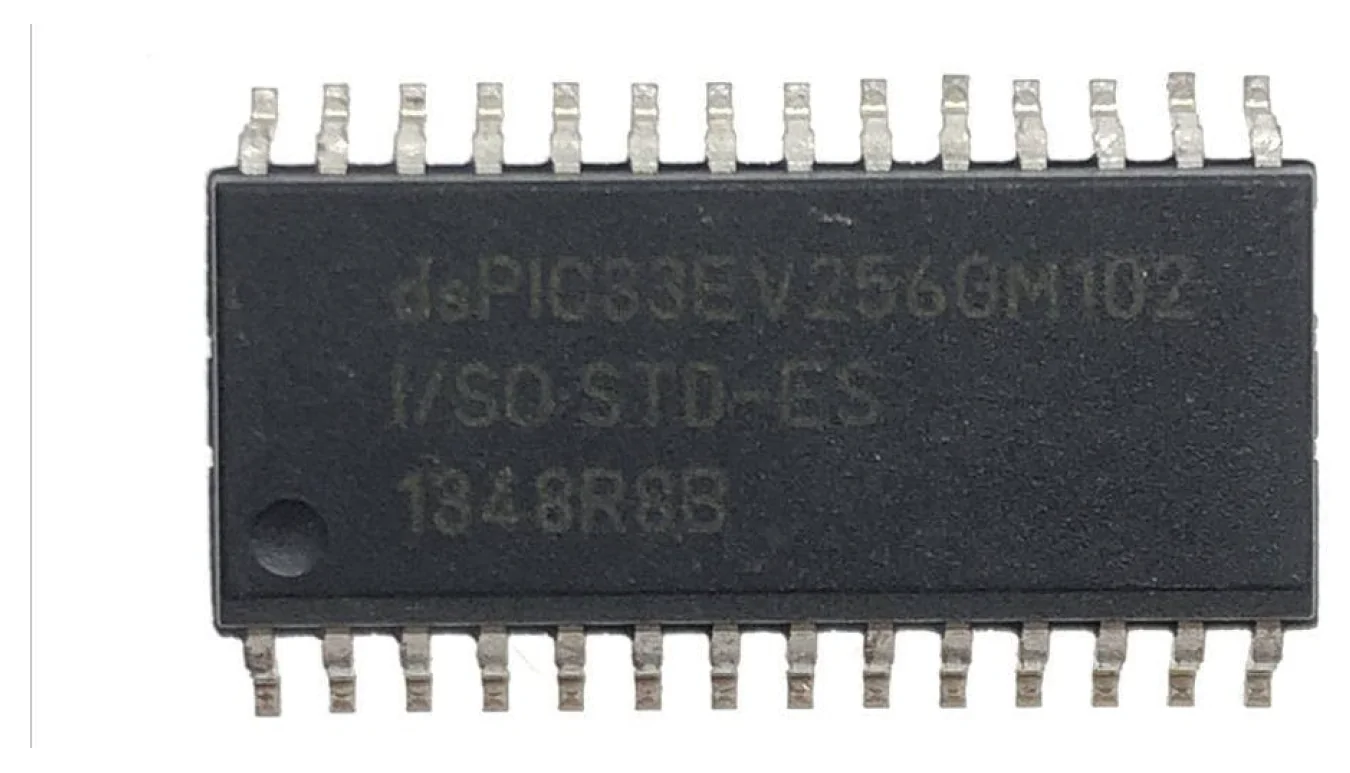
dsPIC33EV256GM102 16-bit microcontroller with embedded 12-bit ADC.

The chosen microcontroller is a 16-bit dsPIC (see Figure 2) with the following:

- Up to 68.75 MHz core frequency (with PLL);
- Multiple 10-bit ADC channels and a single 12-bit ADC used for the gyro signal;
- On-board timers and interrupt controllers;
- An SPI module for high-speed communication with nvSRAM.

An external crystal provides the reference clock, which is internally multiplied via Phase Locked Loop (PLL) to achieve the desired CPU and peripheral timing. The ADC is configured in single-channel, single-conversion mode, triggered within the timer interrupt routine.

#### 4.2.3. Non-Volatile Memory (CY14X101Q2A nvSRAM 2 Click)

**Figure 3:**
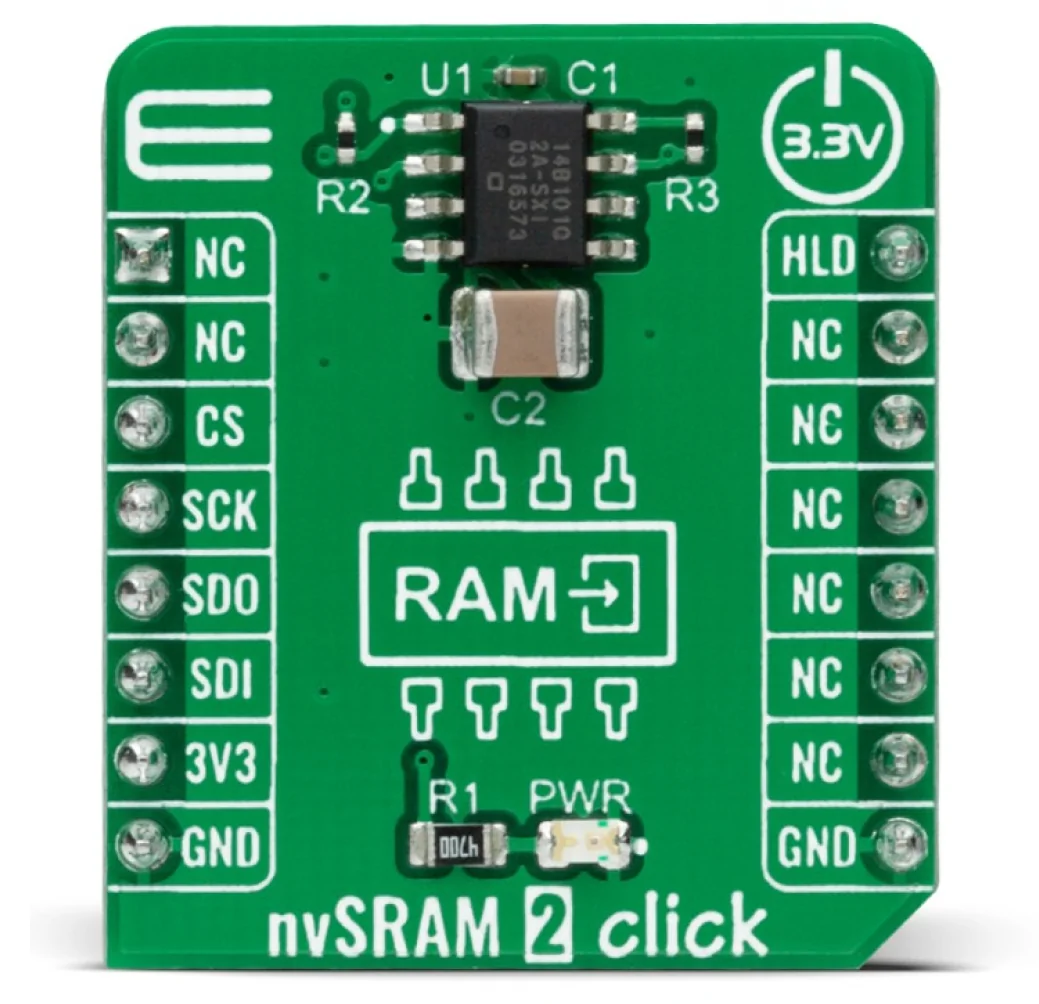
nvSRAM 2 click non-volatile memory.

Data logging is handled by a 1-Mbit nvSRAM 2 Click (128 k × 8-bit) (see Figure 3). This device combines the speed of SRAM with non-volatile storage characteristics, providing the following:

- Byte-addressable read/write over SPI at high speed;
- Automatic store on power loss and recall at power-up;
- Operation from 3.3 to 5 V, making it compatible with the system supply.

The microcontroller uses SPI transactions to stream each new sample (or sample packet) directly into a predefined address range in the nvSRAM. The storage depth and logging duration are determined by the chosen sampling rate and data format.

#### 4.2.4. Power, PCB, and Interfaces

The gyro card includes regulated power rails, decoupling capacitors, and basic protection circuitry to support operation from a single external supply. Indicators (LEDs) provide visual feedback of power and system status, and connectors are provided for the following:

- Power input;
- Optional UART interface (for streaming data during lab work);
- In-circuit programming of the dsPIC.

The PCB development process followed a standard electronic hardware design workflow. It began with the creation of a detailed schematic that captured all functional blocks of the system, including the gyroscope interface, signal conditioning circuitry, power regulation stages, memory subsystem, microcontroller connections, and programming interfaces. Following schematic verification and electrical rule checking, component footprints were assigned, and the board layout was developed. Particular attention was given to component placement, signal routing, grounding strategy, and separation of analog, digital, and power domains to minimize noise coupling and ensure reliable operation. After completing the layout, Design Rule Checks (DRCs) and manufacturing validations were performed, followed by the generation of Gerber files and drilling data required for fabrication. The PCB prototypes were then manufactured in-house using a multilayer LPKF prototyping machine, enabling rapid fabrication, testing, and iterative refinement of the design prior to final system integration and validation.

**Figure 4:**
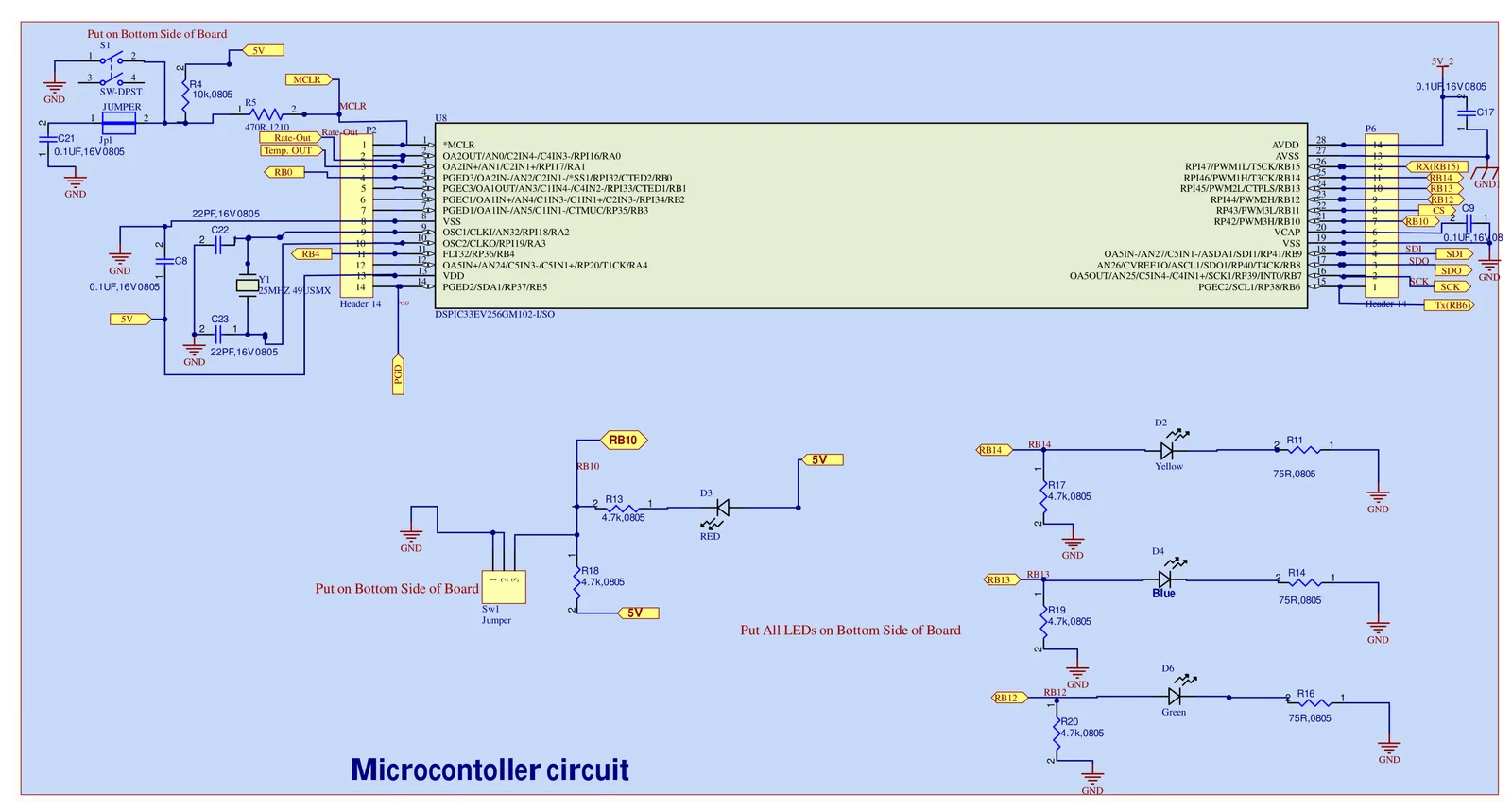
MCU circuit.

**Figure 5:**
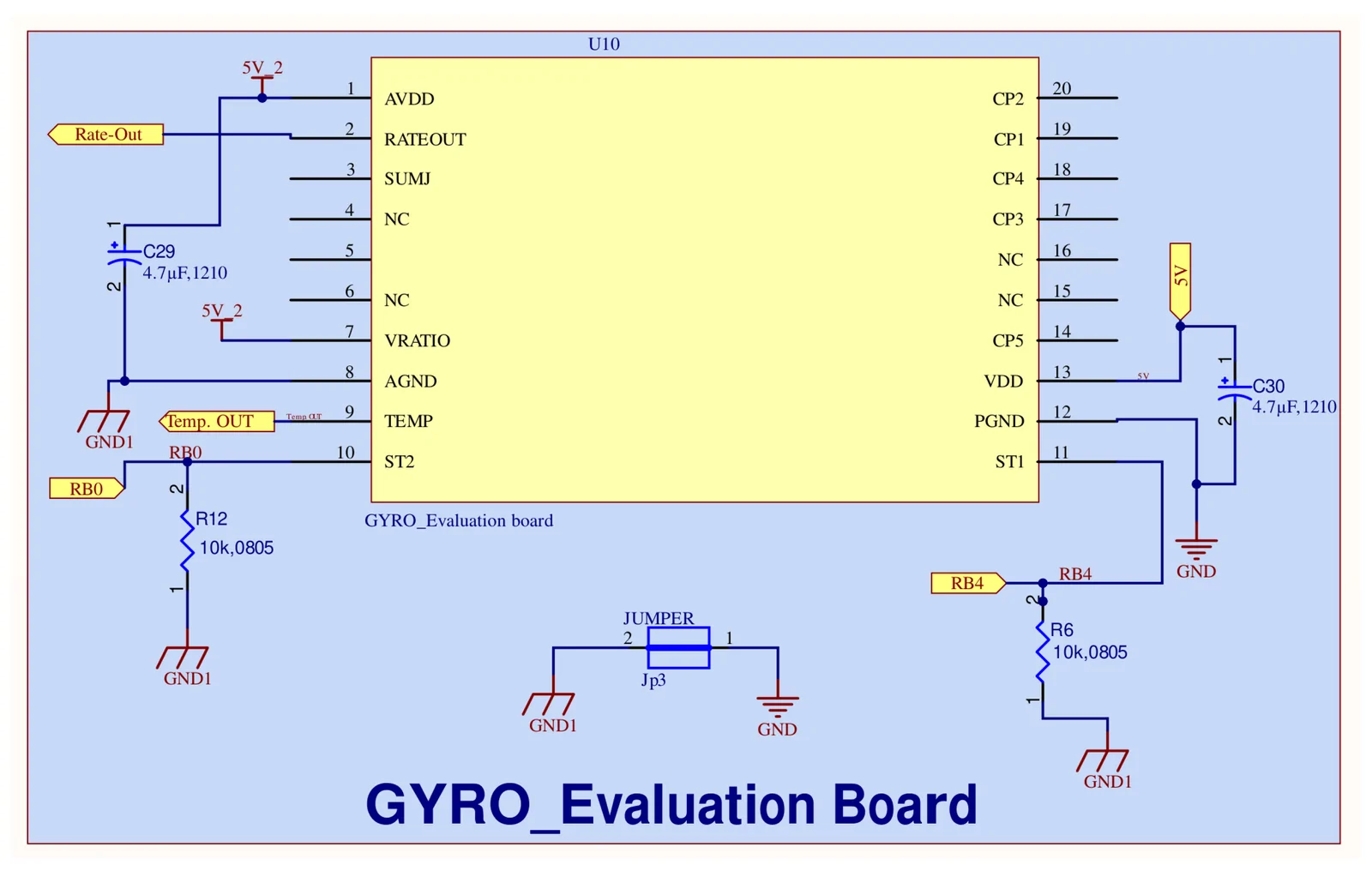
Gyroscope circuit.

**Figure 6:**
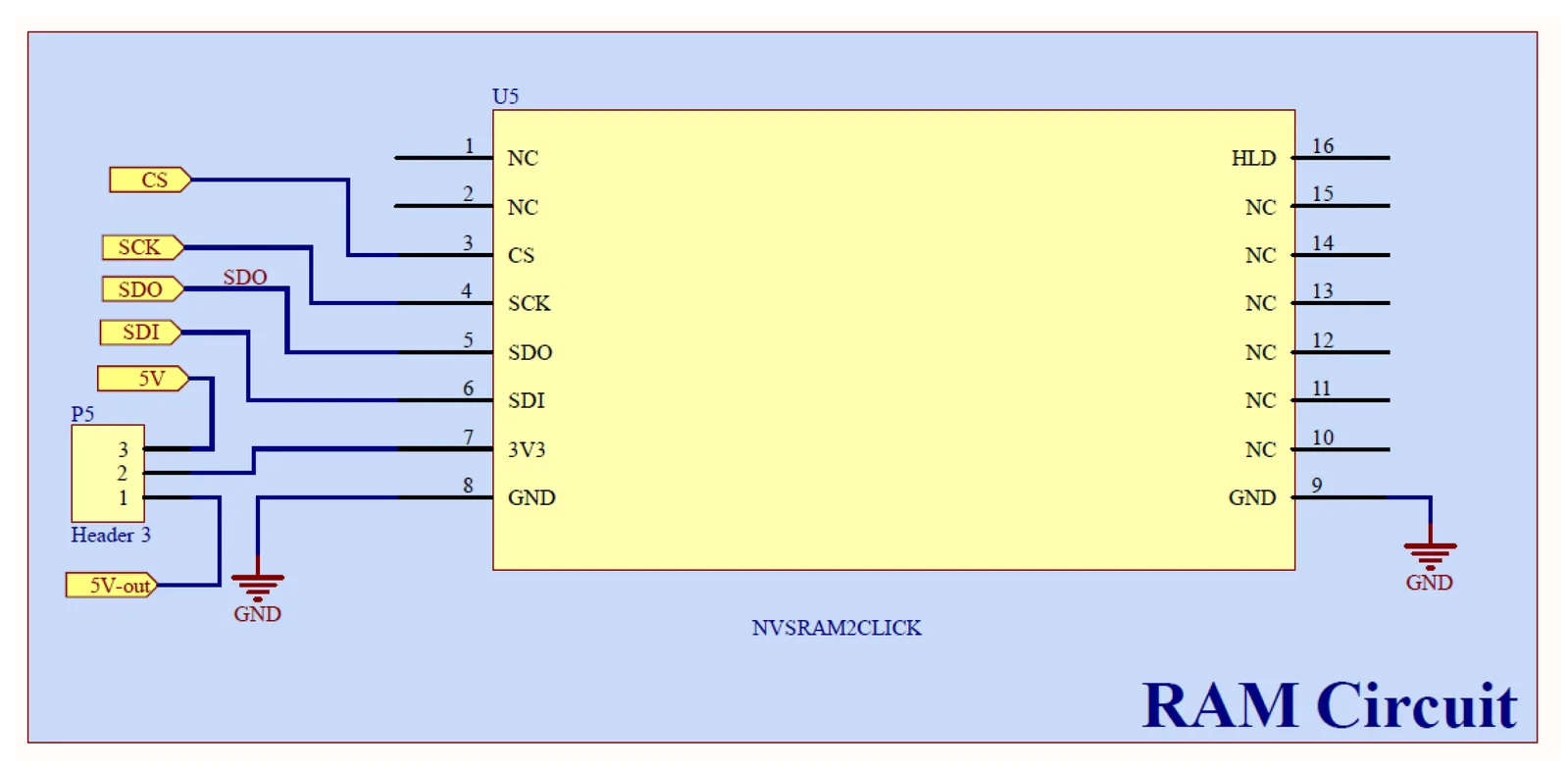
NVSRAM circuit.

**Figure 7:**
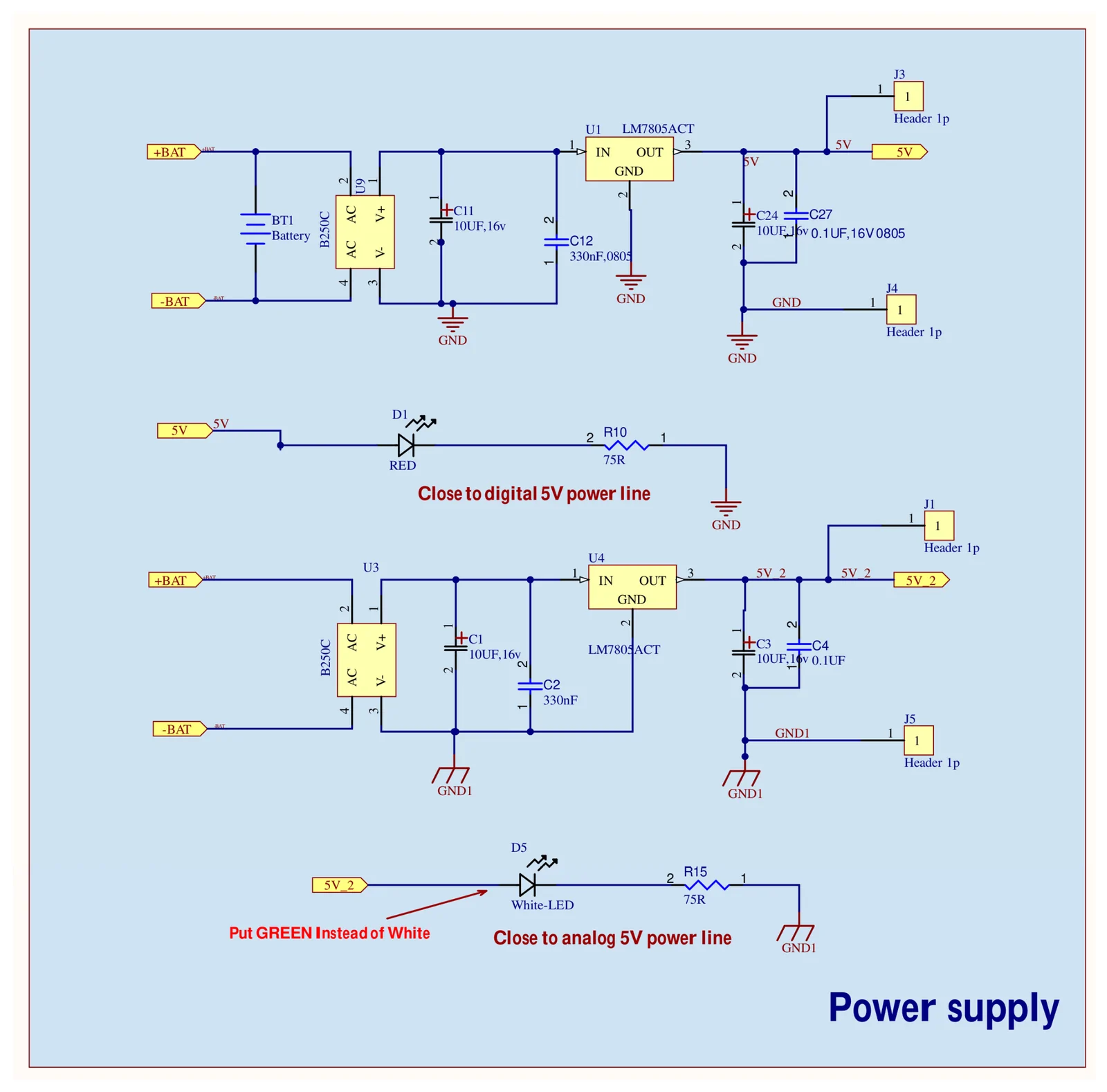
Power supply circuit.

**Figure 8:**
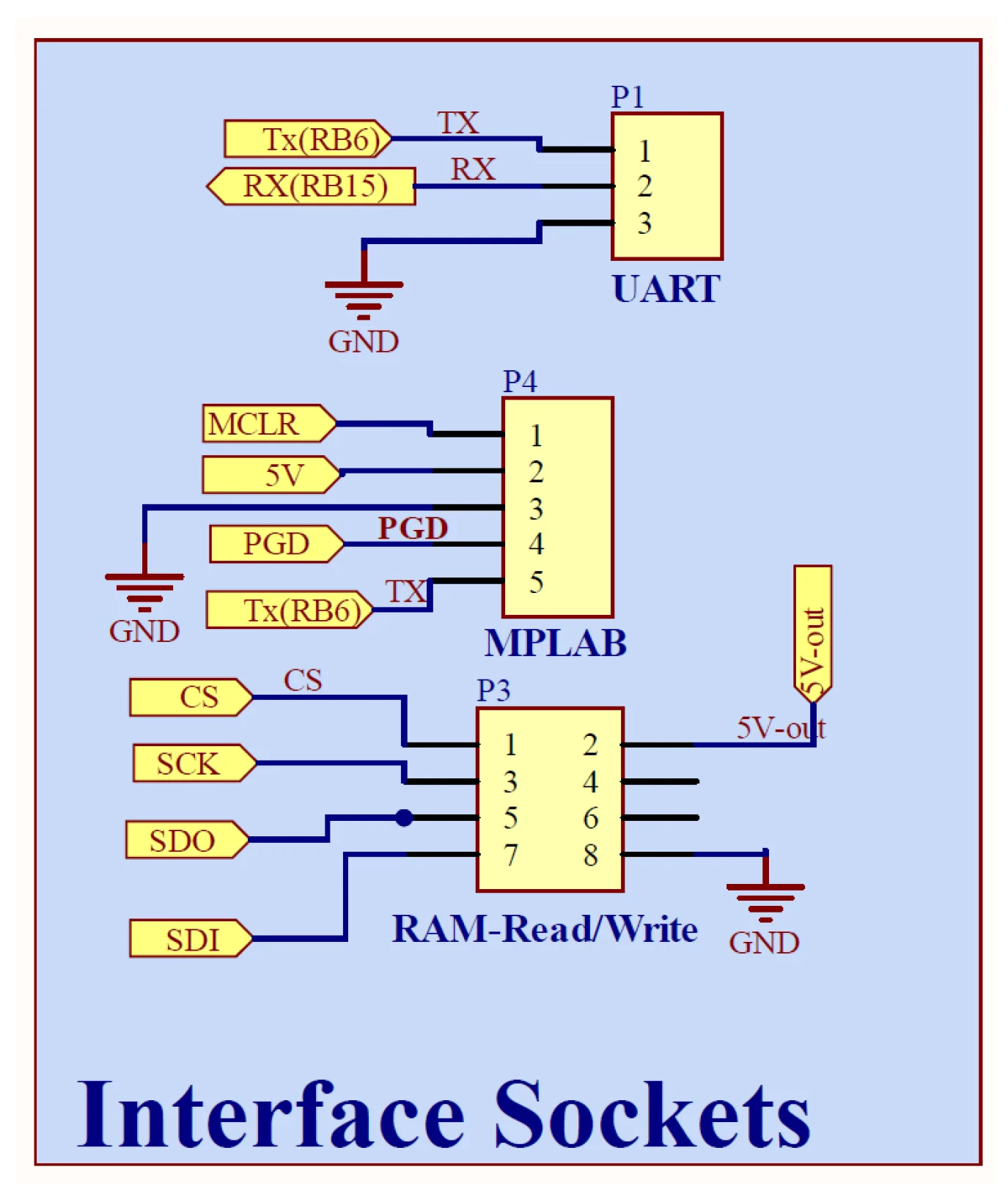
Interface sockets.

**Figure 9:**
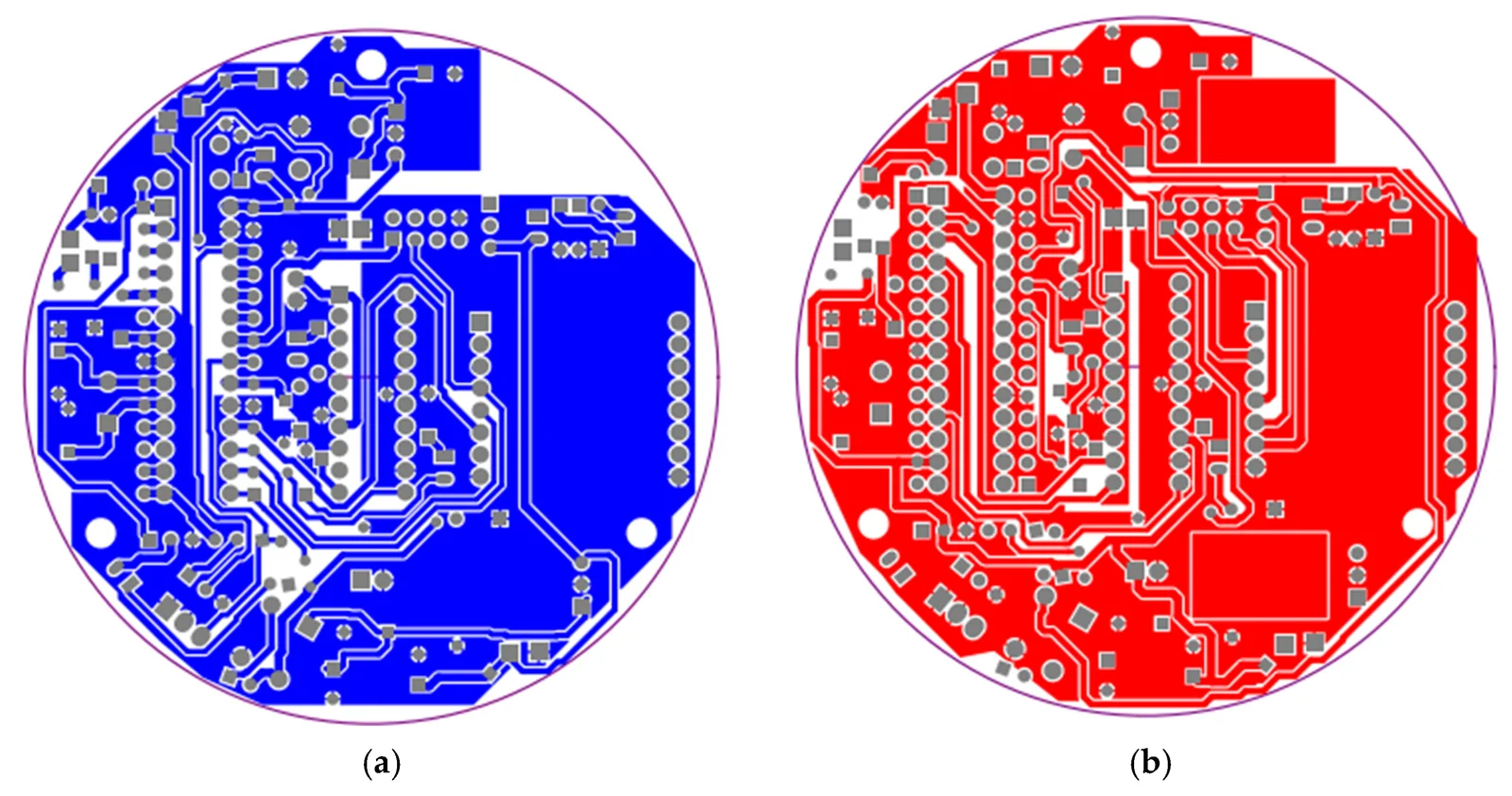
PCB (**a**) solder layer and (**b**) top layer.

**Figure 10:**
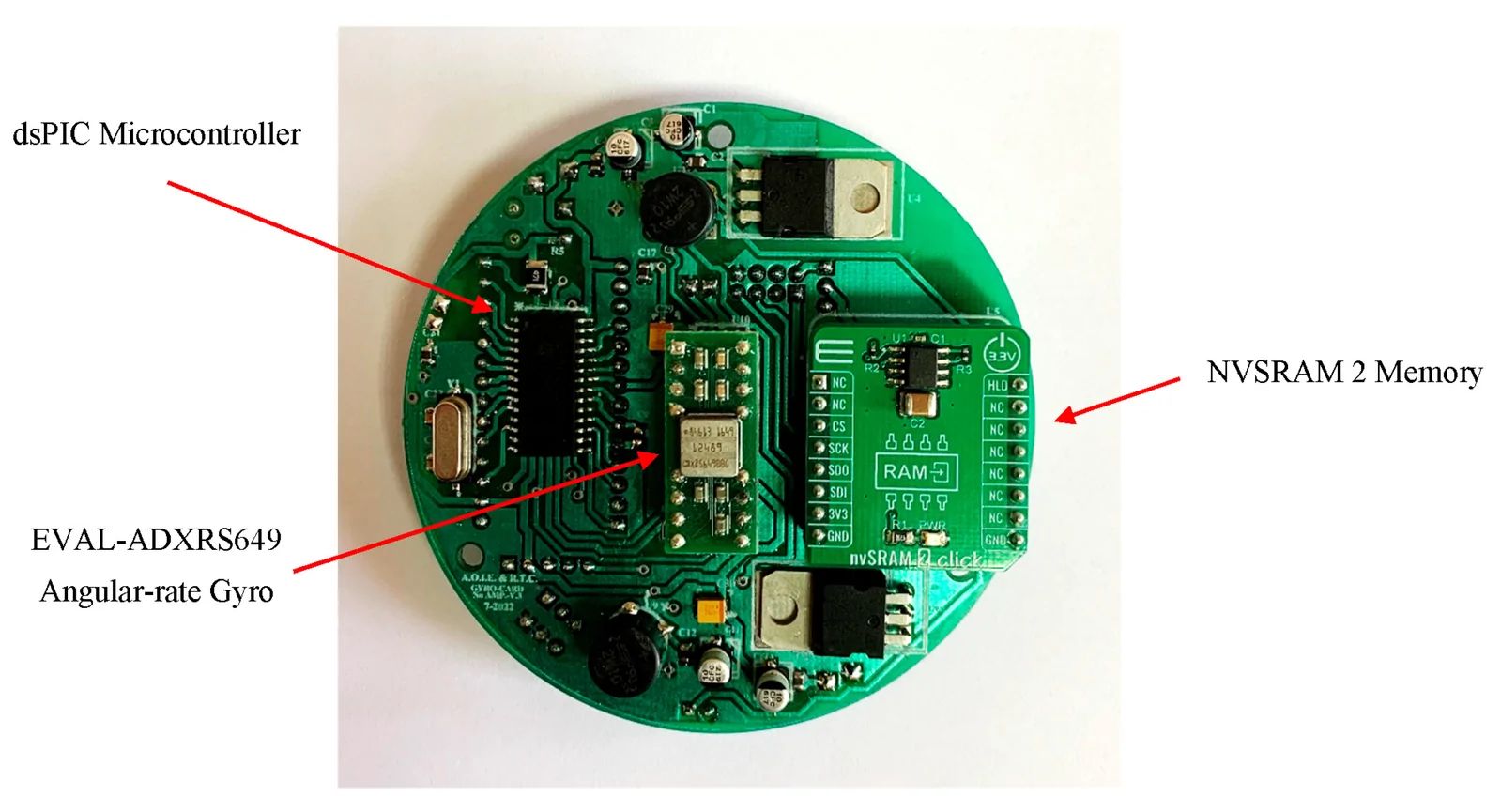
PCB—top layer with components.

**Figure 11:**
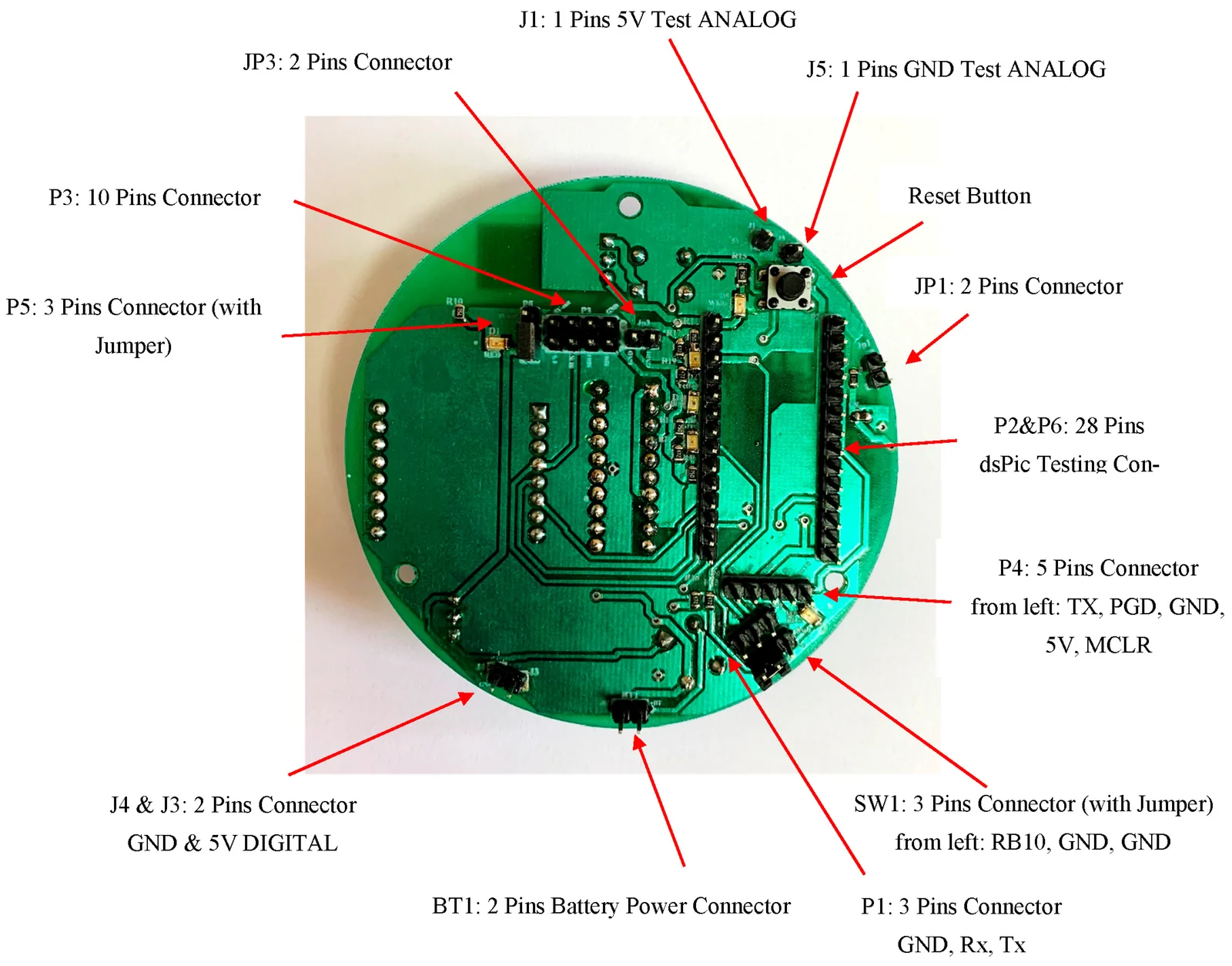
PCB—Bottom Layer with Components.

The detailed schematic, top-layer layout, bottom-layer layout, and the final assembled PCB after component integration are presented in the following figures (Figure 4, Figure 5, Figure 6, Figure 7, Figure 8, Figure 9, Figure 10 and Figure 11) to illustrate the complete hardware development process from schematic capture to fully operational hardware implementation (Note: *SSI the asterisk indicates active-low logic).

### 4.3. Firmware and Data Acquisition Logic

The embedded firmware is structured around a clear initialization–self-test–logging sequence:

#### 4.3.1. Initialization and Self-Test Sequence

The firmware follows a deterministic initialization–verification–logging sequence to ensure that no data are recorded before the sensing and storage chain has been fully validated.

Immediately after power-up, the dsPIC configures all required peripherals while keeping the acquisition path disabled. First, the system clock is initialized: an external crystal is used as a reference, and the internal PLL is configured to achieve a CPU frequency of 68.75 MHz, providing sufficient timing resolution for both ADC conversion and SPI transactions. Next, the SPI module is set up for high-speed communication with the nvSRAM, followed by configuration of the dedicated 12-bit ADC channel connected to the ADXRS649 analog output. A hardware timer is then programmed to define the sampling interval; for example, a period of 20 ms corresponds to a 50 Hz angular-rate update rate. At this stage, the ADC, timer, and their associated interrupts remain configured but disabled, so that no samples are taken until the system has passed its internal health checks.

Once the peripheral configuration is complete, the firmware performs a memory integrity check on the nvSRAM. Two known bytes are written to the first memory addresses over SPI and then read back. Successful comparison confirms that both the memory device and the SPI interface are functioning correctly and that non-volatile storage is available for logging. If the comparison fails, the system flags a fault condition and does not proceed to normal operation.

The next step is a gyroscope self-test, consistent with the recommendations for the ADXRS649. The sensor provides two self-test pins, ST1 and ST2, which inject known internal stimuli corresponding to fixed angular-rate offsets. The firmware activates ST1 and collects a fixed number of samples (e.g., 100 readings), then deactivates ST1, activates ST2, and collects another 100 readings. For each self-test condition, the mean ADC code is computed. The difference between the ST1 and ST2 responses is then evaluated against the nominal self-test behavior specified by the manufacturer through a “self-test mismatch” criterion. Only if this mismatch lies within a predefined tolerance band is the gyroscope declared healthy and suitable for use.

After both the memory and gyroscope checks have passed, the acquisition chain is armed. The ADC is enabled and primed to sample the gyro output, the hardware timer is started, and the timer interrupt is activated. From this point onward, the system operates in an interrupt-driven mode: at each timer overflow (for example, every 20 ms in the 50 Hz configuration), the interrupt service routine triggers an ADC conversion of the gyro output, retrieves the resulting 12-bit digital code, and writes it to the next available address in the nvSRAM via SPI. The write pointer is incremented after each sample, creating a continuous, time-ordered log of angular-rate measurements.

This sequence cleanly separates initialization and health verification from normal data-logging operation, ensuring that the recorded high-spin angular-rate data originate from a verified sensor–memory chain and are sampled at a well-defined, constant interval.

#### 4.3.2. Theory of Operation

The overall operation of the gyro card can be summarized as a sequence in which the analog angular-rate signal is digitized, verified, and then logged to non-volatile memory at a fixed sampling rate. The ADXRS649 produces an analog voltage proportional to the applied angular rate. This voltage is sampled by the on-chip 12-bit ADC of the dsPIC, processed by the microcontroller, and then written to the nvSRAM at each sampling instant.

At the beginning of each power cycle, no data is recorded until the integrity of the storage and sensor has been confirmed. The microcontroller first performs a simple memory handshake: two known bytes are written to the first locations of the nvSRAM and then read back over SPI. Successful comparison indicates that the memory device and interface are functioning correctly and are ready to be used as a logging medium.

The gyroscope is then verified using its built-in self-test functionality. The ADXRS649 provides two self-test pins, ST1 and ST2, which apply internal electrostatic actuation to emulate nominal angular-rate offsets of approximately −1300°/s and +1300°/s, respectively. For each self-test state, the firmware collects a block of samples and computes the average digital output, denoted ST1 and ST2 for the two excitation cases. Following the manufacturer’s recommendation, a self-test “accuracy” metric is computed as

(1) $$\mathrm{accuracy}=\frac{ST2+ST1}{(ST2-ST1)/2}$$

Ideally, the numerator should be small compared with the denominator, and the resulting ratio should lie within a specified tolerance band. To make the above formula clearer, the equation quantifies the normalized asymmetry between the two internal self-test responses generated by the ADXRS649. In an ideal sensor, the responses produced by ST1 and ST2 are equal in magnitude and opposite in sign, resulting in an accuracy value of zero. The numerator represents the deviation from this ideal symmetry, while the denominator normalizes this deviation by the average self-test amplitude. Consequently, the metric provides a dimensionless measure of the self-test imbalance. There is no unique pair of ST1 and ST2 values corresponding to an accuracy of ±2%. As a numerical example, suppose the self-test amplitudes are close to their nominal values, i.e., ST1 = −1300, ST2 = 1300. Then, according to the above formula, the accuracy = 0% (which represents a perfectly balanced sensor). Suppose that ST1 = −1290, ST2 = 1310. Then, the accuracy = 1.54%, which satisfies the ±2% criterion. Moreover, suppose that ST1 = −1270, ST2 = 1330. Then the accuracy = 4.6%, which fails the criterion (for more details regarding the self-test process, see https://www.analog.com/ADXRS649/datasheet (accessed on 12 May 2026)).

In this work, the gyroscope is considered healthy if the value of (1) remains within ±2% of the nominal range indicated in the device documentation; otherwise, the sensor is flagged as out of specification, and the logging phase is not started.

Once both the memory and the gyroscope have passed their checks, the firmware enables the timer-driven acquisition. A hardware timer is configured with a period corresponding to the desired sampling frequency (for example, a period of 20 ms yields a 50 Hz update rate). At each timer overflow, the microcontroller enters a timer interrupt service routine, triggers a single ADC conversion on the gyro channel, and obtains a 12-bit digital value d∈[0,4095]. The full-scale analog range is mapped as follows:

- d=0 corresponds to approximately $-{20,000}^{\circ }/s$;
- d≈2048 corresponds to $0^{\circ }/s$;
- d≈4095 corresponds to $+{20,000}^{\circ }/s$.

Each converted sample is then written to the next available address in the nvSRAM via SPI, and the write pointer is incremented. This process yields a time-ordered sequence of angular-rate measurements acquired at a constant, well-defined sampling interval and originating from a sensor–memory chain that has been explicitly verified at power-up.

#### 4.3.3. Memory Integrity Check

Before any measurement is accepted as valid, the microcontroller performs a handshake test with the nvSRAM:

- Two known bytes are written to the first addresses;
- The same locations are read back and compared.

If a mismatch is detected, the system flags a memory fault and does not proceed to normal logging.

#### 4.3.4. Gyroscope Self-Test and Health Check

The ADXRS649 provides internal self-test pins (ST1, ST2) that simulate known angular-rate offsets when activated. The firmware performs as follows:

- Collects a set of samples (e.g., 100) with ST1 active;
- Collects another set with ST2 active;
- Computes the mean digital output in each condition;
- Evaluates a “self-test mismatch” against the nominal response specified by the manufacturer.

If the measured response lies outside an acceptable tolerance band, the gyro is considered out of specification, and data logging is inhibited.

#### 4.3.5. Timer-Driven Sampling and Logging

After both memory and gyro have passed their checks, the system enables the following:

- The 12-bit ADC;
- The configured hardware timer;
- The global interrupt that services timer overflows.

At each timer overflow, the following occur:

- An Interrupt Service Routine (ISR) is entered;
- The ADC samples the gyro output and performs a conversion;
- The resulting 12-bit code (0–4095) is packaged (e.g., into a 16-bit word or a small frame with an index) and written to nvSRAM via SPI;
- The write address is incremented for the next sample.

The mapping between ADC code and angular rate follows a linear relationship:

- 0 → −20,000°/s;
- 2048 → 0°/s;
- 4095/4096 → +20,000°/s.

**Figure 12:**
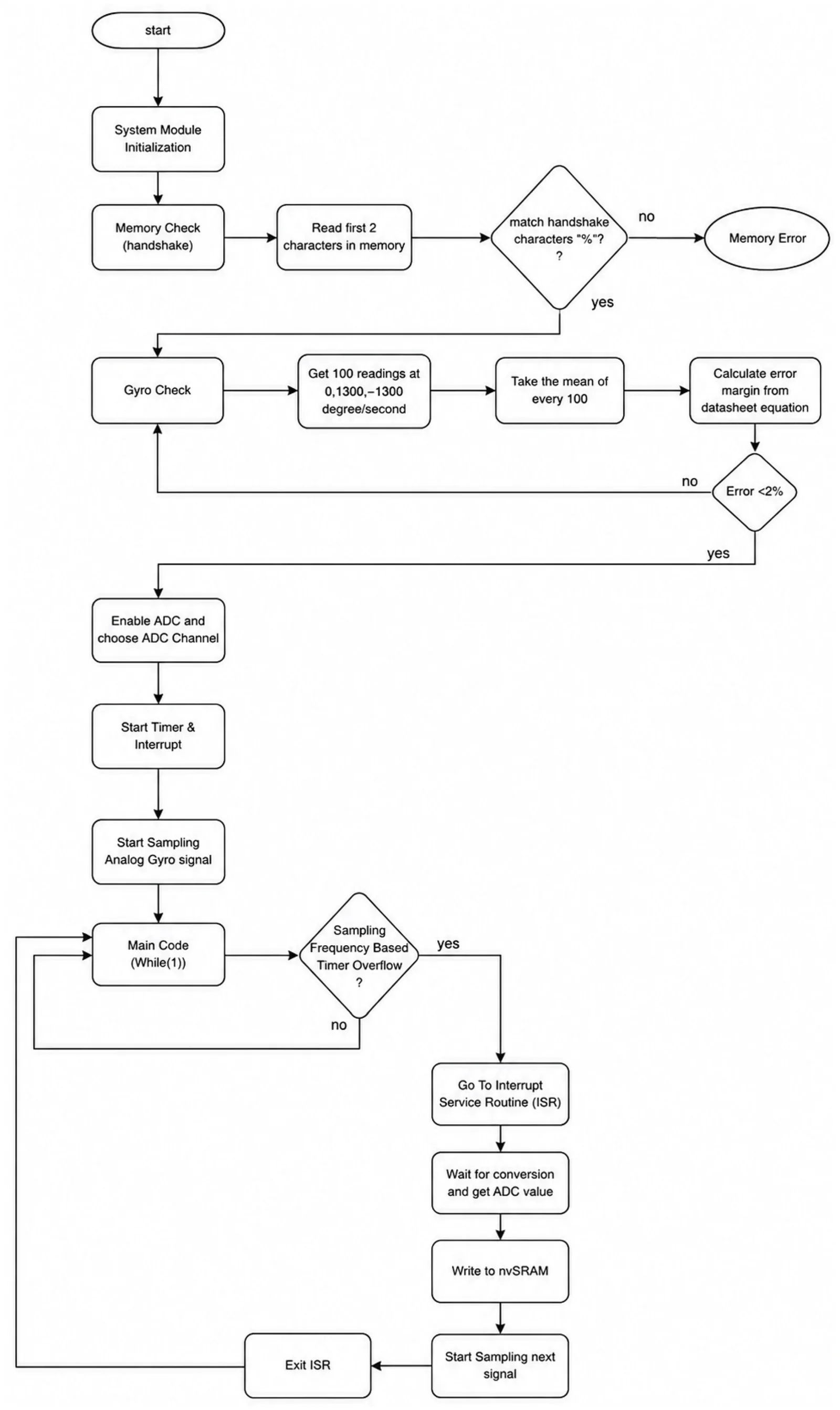
Firmware flowchart.

This mapping is later refined by the calibration process, which provides more accurate bias and scale-factor estimates. The overall sequence of the firmware structure is summarized in the flowchart in Figure 12.

#### 4.3.6. Optional UART Streaming for Lab Use

For laboratory characterization and debugging, the same firmware base can be compiled in a UART mode, where the ADC samples are sent directly to a PC (e.g., via PuTTY) instead of—or in addition to—being stored in nvSRAM. This facilitates rapid visualization and post-processing in MATLAB R2016b without exhausting on-board memory.

### 4.4. Laboratory Calibration and Testing Strategy

To ensure that the gyro card delivers quantitatively reliable angular-rate measurements, we designed a calibration and testing strategy comprising both static and dynamic experiments. It is important to highlight that the purpose of the deterministic calibration is not to establish a complete inertial error model but rather to estimate the dominant deterministic errors (bias and scale factor) required for accurate operation of the proposed data acquisition system.

#### 4.4.1. Mechanical Setup

**Figure 13:**
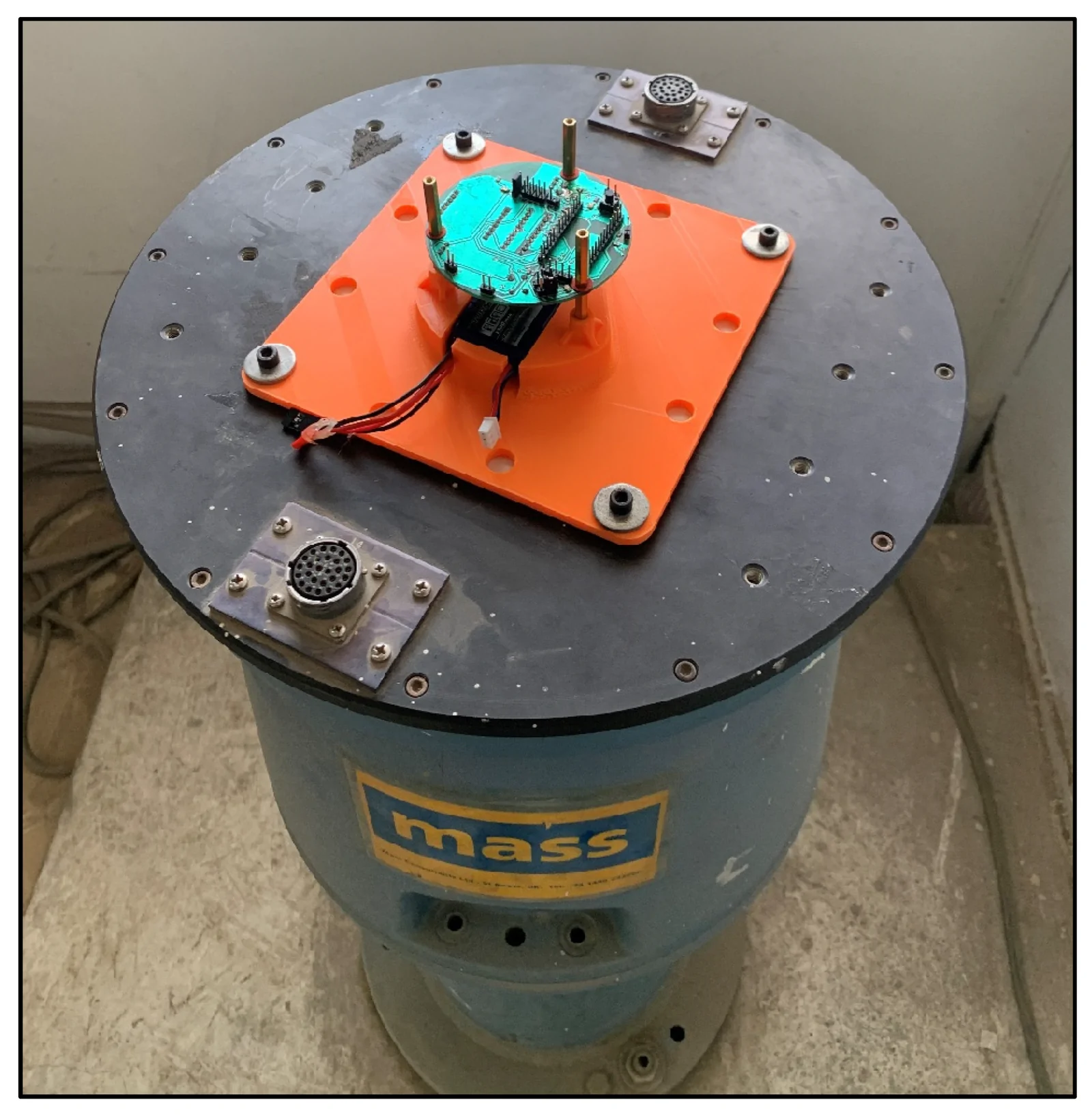
Gyro PCB on test fixture mounted on laboratory turntable.

A single-axis precision turntable is used as the reference rate source. The gyro card is mounted in a custom 3D-printed fixture (appears as orange flat surface in Figure 13) that aligns the sensitive axis of the ADXRS649 with the rotation axis of the turntable, minimizing misalignment errors. This fixture also ensures reproducible orientation across multiple test runs. The custom 3D-printed mounting fixture was designed to provide rigid and repeatable positioning of the gyro card on the tabletop of the turntable while minimizing alignment errors between the sensing axis and the turntable rotation axis. Since the proposed system employs a single-axis analog gyroscope rather than a multi-axis IMU, and all calibration experiments were performed under controlled single-axis rotation, multi-axis cross-coupling compensation was not required.

#### 4.4.2. Static Tests

Static tests are performed with the turntable at rest (0°/s). The card records data over a sufficiently long interval to achieve the following:

- Estimate the bias (mean output at zero rate);
- Characterize short-term noise behavior (standard deviation, qualitative drift patterns).

These zero-rate datasets form the baseline for subsequent dynamic calibration.

#### 4.4.3. Dynamic Calibration: Clockwise (CW) and Counter-Clockwise (CCW) Scenarios

Dynamic calibration involves applying known constant rotation rates in both CW and CCW directions to achieve the following:

- Estimate the scale factor relating ADC output to true angular rate;
- Verify the linearity of the gyro response over the operating range used;
- Assess repeatability and symmetry between CW and CCW rotations.

Turntable rotation scenarios were conducted at different angular rates covering its full operating range of (−980 to +980 deg/s) as follows:

##### CCW

−980, −960, −940, −920 deg/s (20 min total, 5 min at each rate);
−900, −650, −400, −150 deg/s (20 min total, 5 min at each rate);
−65, −45, −25, −5 deg/s (20 min total, 5 min at each rate).

Static Condition

- 0 deg/s (20 min).

##### CW

- 5, 25, 45, 65 deg/s (20 min total, 5 min at each rate);
- 150, 400, 650, 900 deg/s (20 min total, 5 min at each rate);
- 920, 940, 960, 980 deg/s (20 min total, 5 min at each rate).

The selected measurement durations represent a compromise between statistical estimation accuracy and practical calibration time. At a sampling frequency of 50 Hz, each 5-min constant-rate experiment yields approximately 15,000 samples, while the 20-min static test provides approximately 60,000 samples for bias estimation. Averaging over such large datasets improves the confidence of the estimated mean angular rate. In other words, the calibration estimates are obtained from 15,000 samples per operating point. Increasing the acquisition time beyond the selected intervals would further reduce the variance of the mean estimate; however, the improvement becomes progressively smaller because the remaining uncertainty is primarily governed by deterministic calibration errors, including bias and scale-factor errors. Therefore, the adopted measurement durations were considered sufficient to obtain stable and repeatable calibration parameters while maintaining a practical experimental procedure.

The aforementioned rotation scenarios were executed, and the gyroscope measurements were continuously recorded. The recorded data were subsequently extracted and processed after the completion of each test scenario. For each scenario, the measured angular rate values (after calculating their mean values) were plotted and compared against the corresponding turntable reference angular-rate values to evaluate the gyroscope performance and calibration accuracy.

Mathematically, a linear model is adopted for each dataset:

(2) $$\omega_{true}=S_{f}(d-d_{0})$$

where

- $\omega_{\mathrm{true}}$ is the known turntable rate;
- d is the measured digital output;
- $d_{0}$ is the bias term estimated from static tests;
- $S_{f}$ is the scale factor to be identified.

**Figure 14:**
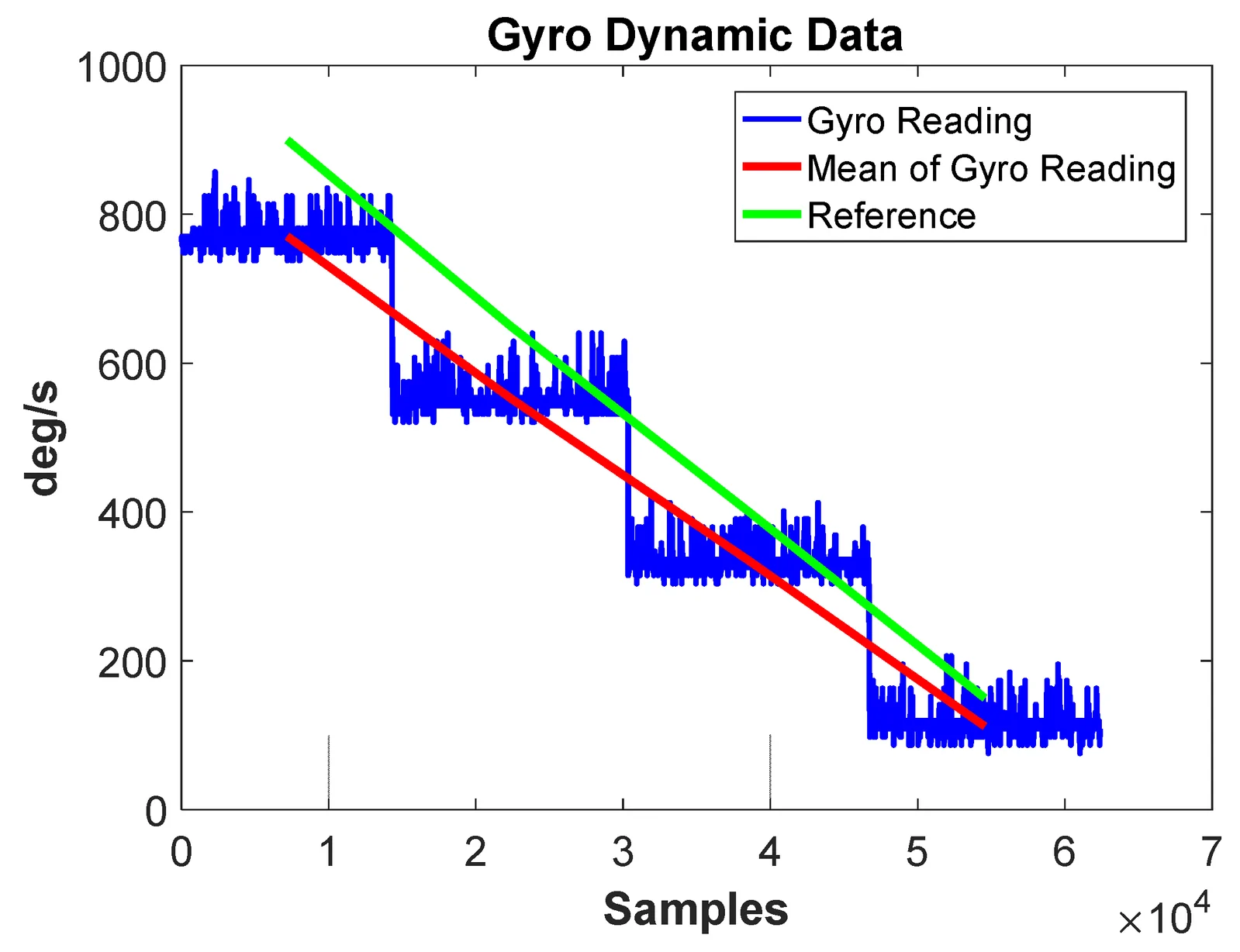
Gyro dynamic data output (900 to 150 deg/s) with reference (turntable feedback rotational rate).

By aggregating results over multiple CW/CCW runs, we obtain robust estimates of $d_{0}$ and $S_{f}$, along with measures of their variability, which directly quantify the calibration quality of the gyro card (see Figure 14).

**Figure 15:**
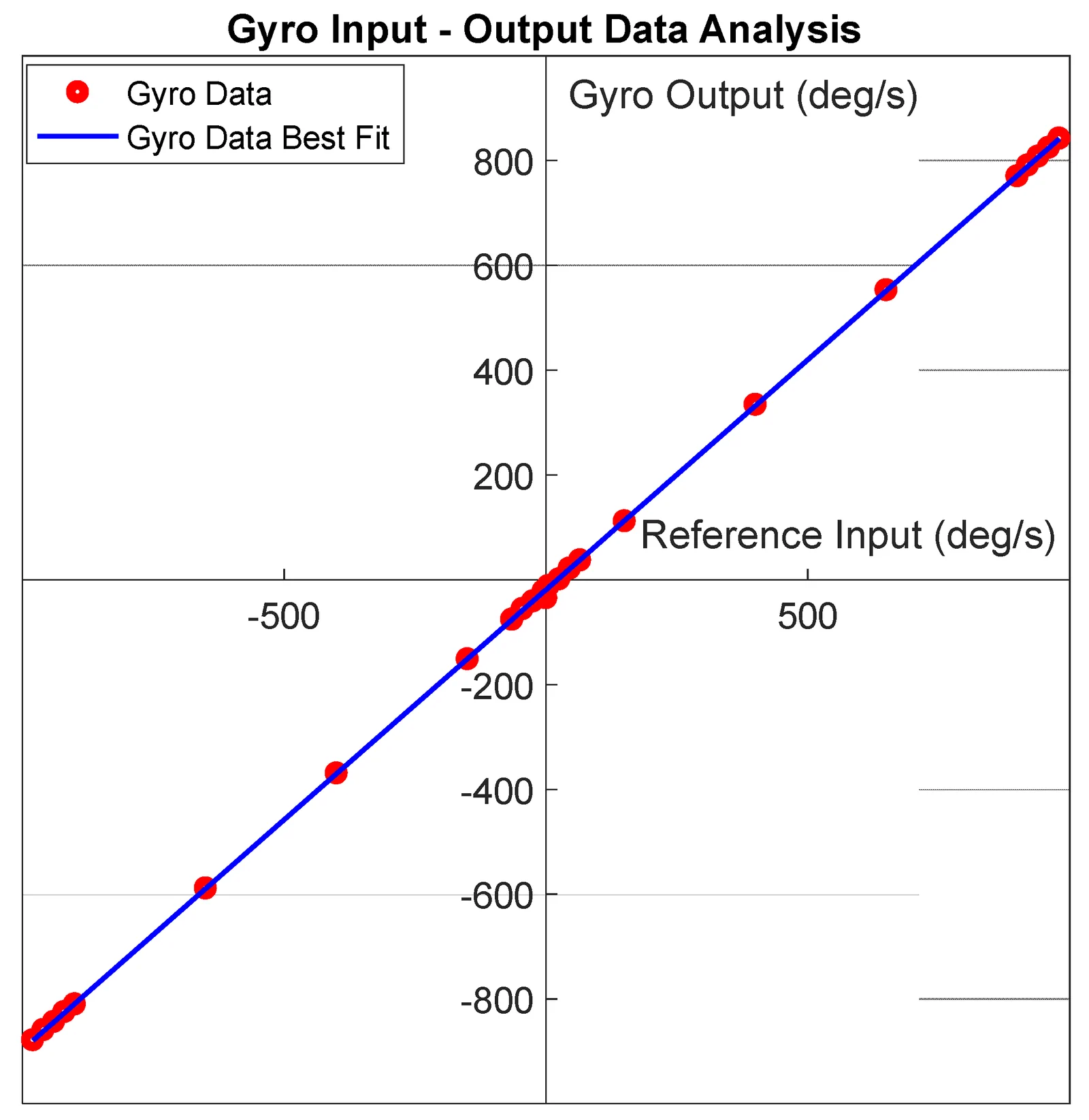
Relation between gyro input (turntable rate feedback) and gyro output data.

The relationship between the reference input (turntable angular rate feedback) and the gyroscope output was plotted as shown in Figure 15. A first-order linear fit was then applied to the data to characterize the sensor response and to facilitate the estimation of the gyroscope’s linear error parameters, including scale-factor error, bias (offset), and linearity performance.

The linear error parameters (bias and scale factor (SF)) were estimated from the previously established relationship between the reference input and the gyroscope output:

- Bias = −18.87 deg/s, (95% confidence interval: −20.29 to −17.27 deg/s);
- Scale Factor (SF) = 0.878, (95% confidence interval: 0.8756 to 0.8803).

The mean gyroscope measurements were subsequently corrected using the estimated linear error parameters. The corrected angular-rate measurements were then compared with the corresponding turntable reference values to assess the effectiveness of the calibration process and the resulting measurement accuracy.

According to the results in Table 3, it is worth noting that the largest relative errors are observed at the lowest angular rates (e.g., ±5°/s). This behavior is expected because, at very low input rates, the measured signal becomes comparable to the residual bias, sensor noise, ADC quantization error, and other low-level disturbances. Consequently, even a small absolute measurement error results in a relatively large percentage error due to the small magnitude of the reference angular rate. In contrast, the same absolute error represents only a small fraction of the measured value at higher angular rates. Furthermore, the bias and scale-factor coefficients were estimated using a first-order linear fit calibration procedure over the entire operating range (−980°/s to +980°/s), which minimizes the overall error across all calibration points rather than optimizing the estimation accuracy at very low angular rates. Moreover, it should be noted that percentage error is not an appropriate performance metric for angular rates close to zero because the small denominator artificially inflates the relative error. For low-rate measurements, absolute error, bias stability, and noise characteristics provide a more representative assessment of sensor performance. Since the main target of the proposed DAS is to measure high angular rates, the relatively large errors observed at low angular rates (e.g., ±5°/s) could be neglected.

To provide a quantitative assessment of the proposed deterministic calibration, several statistical performance metrics were computed from the residual calibration errors (see Table 4). Across all calibration points, the proposed method achieved a mean error of approximately 0 deg/s, confirming the absence of systematic bias after calibration. The overall RMSE and standard deviation were 3.99 deg/s, primarily influenced by the residual error at the zero-rate measurement. Excluding the static operating point, which mainly reflects residual bias, the dynamic calibration exhibited an RMSE of 1.63 deg/s and a maximum absolute error of 3.06 deg/s over the investigated angular-rate range. The low MAE and RMSE values further demonstrate that the calibrated measurements closely follow the turntable reference over the investigated dynamic range. Although the residual error at the static operating point (0°/s) slightly increases the overall statistical metrics due to the remaining zero-rate bias, the dynamic calibration results exhibit low residual errors and confirm the effectiveness and repeatability of the proposed calibration methodology.

It is important to mention that the calibration procedure presented in this work focuses on compensating the dominant deterministic error sources of the analog gyroscope, namely bias and scale-factor errors. These parameters have the greatest influence on measurement accuracy for the intended high-spin, short-duration operating scenarios. Although stochastic error characteristics such as ARW and BI provide valuable information for long-duration inertial navigation systems and are typically estimated using, for instance, Allan variance analysis, they were not investigated in this study because the proposed DAS is intended for relatively short-time missions (this refers to the available data logging time of the selected nvSRAM memory associated with a 50 Hz sampling rate). Such hardware could log angular rate measurement data of approximately 20 min, during which deterministic calibration errors constitute the primary contributors to the overall measurement uncertainty. Moreover, at high angular rates, the signal magnitude is several orders of magnitude larger than the gyroscope’s noise floor. Therefore, for the relatively short measurement interval considered, deterministic calibration errors (bias and scale factor) have a much greater impact on measurement accuracy than stochastic noise processes such as ARW and BI. Consequently, stochastic characterization is considered beyond the scope of the present work and will be addressed in future investigations.

### 4.5. Preparation for Real High-Spinning Tests

Finally, to validate the complete system under realistic operating conditions, the developed gyro card is prepared for high-spin experiments. The preparation process focuses on improving the mechanical and environmental robustness of the integrated system and consists of the following main elements:

1. Rugged Enclosure

**Figure 16:**
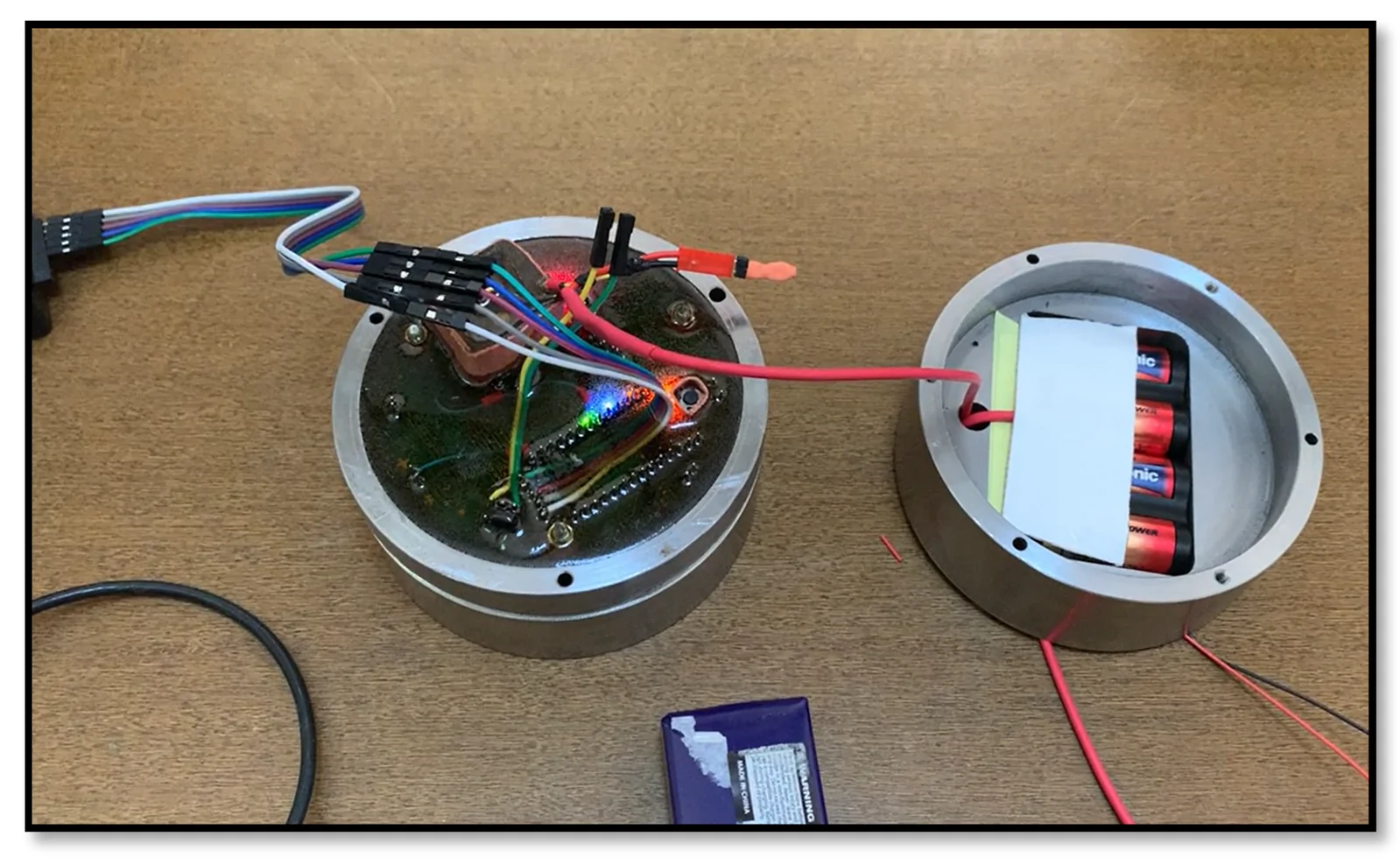
Gyro PCB (after putting) with batteries after installation into a custom metal enclosure.

The PCB is installed inside a custom-designed metallic enclosure capable of withstanding the mechanical stresses associated with high-spin operation (see Figure 16). The enclosure provides structural support, protects the electronics against mechanical shock and vibration, offers Electromagnetic Interference (EMI) shielding, and incorporates the necessary mechanical interfaces for secure integration with the vehicle or laboratory rotary platform.

2. Potting and Environmental Protection

To further enhance system reliability, the complete electronic assembly is encapsulated using a polyurethane potting compound. The potting material mechanically secures the electronic components, reducing susceptibility to vibration-induced fatigue, connector movement, and mechanical stress generated during high-speed rotation. It also provides environmental protection against moisture, dust, and contaminants while helping to reduce rapid localized temperature variations across the electronic assembly, thereby improving the overall stability and reliability of the system under harsh operating conditions. Before and after the potting process, the complete card is powered and tested to verify successful execution of the built-in self-test routines and to confirm that the gyroscope output remains within its nominal operating characteristics.

**Figure 17:**
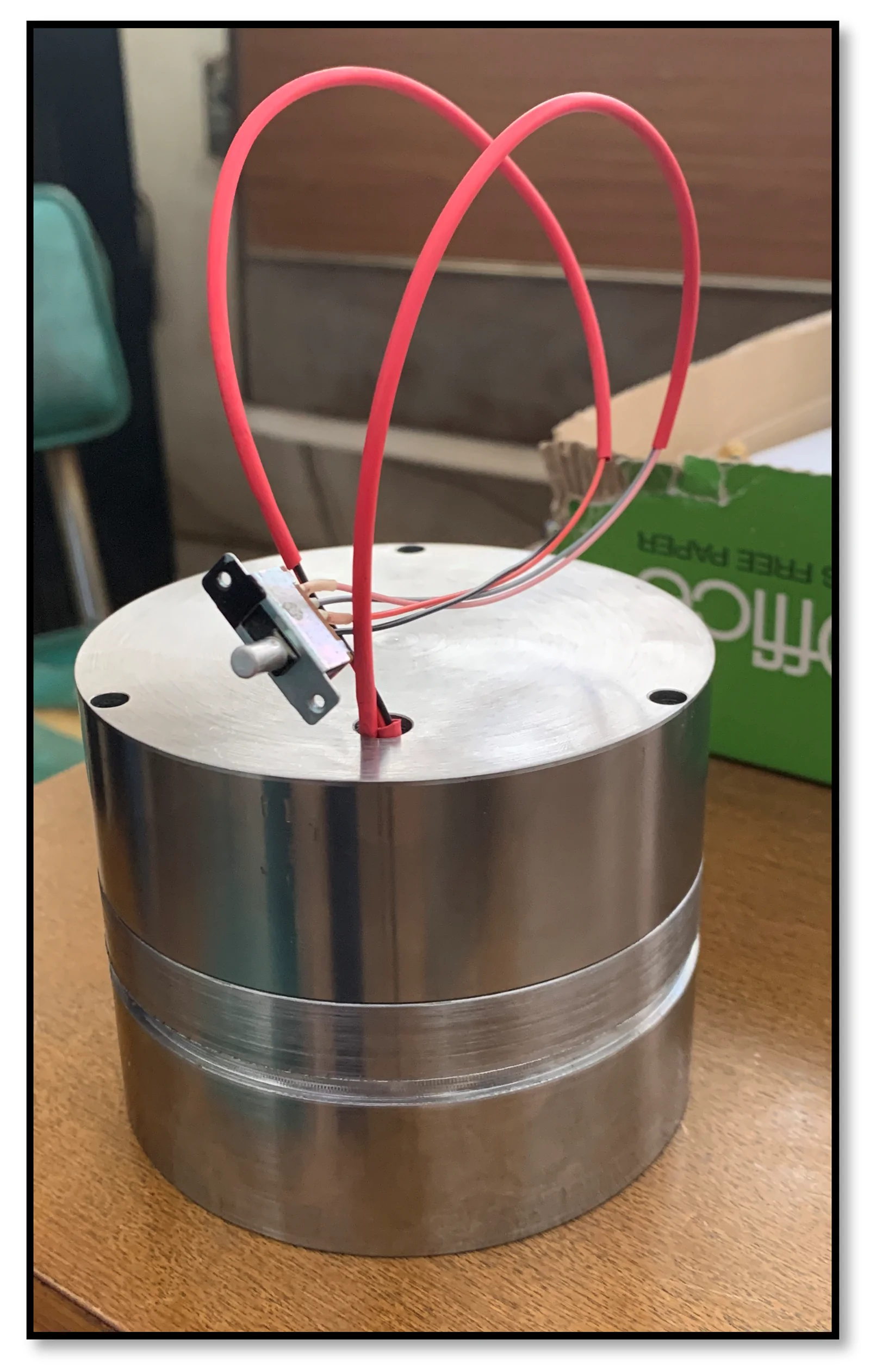
Final custom metal enclosure with external ON/OFF switch.

The final metal enclosure for the gyro PCB with power batteries and external ON/OFF switch is highlighted in the Figure 17.

3. Power Management and Operation Mode

A one-way switch and appropriate wiring are used to power the card shortly before starting the mission. In the mission configuration, the system runs purely in autonomous logging mode, using nvSRAM as storage with no external communication during the mission. After recovery, the stored data are downloaded and processed offline. The corresponding experimental results—both from the laboratory calibration campaigns and from the real in-lab high-spin test—are presented and discussed in the subsequent section.

## 5. Experimental Tests and Results

The proposed angular-rate data acquisition kit was evaluated through a combination of laboratory tests and a real high-spin experiment. The laboratory phase was designed to characterize and calibrate the sensor response under controlled conditions, whereas the in-lab high-spinning rate test was used to verify that the complete, packaged system can survive and operate reliably in a realistic high-spin environment.

### 5.1. Laboratory Testing and Calibration

#### 5.1.1. Static Testing

The first step was to verify the basic behavior of the gyro card under static conditions. The card was powered on a laboratory bench with the sensing axis kept nominally at rest. In this configuration, the system ran in its standard logging mode: after successful memory and self-test verification, the timer-driven interrupt loop sampled the ADXRS649 output at the configured rate and stored the 12-bit samples in nvSRAM.

Static datasets were then extracted and post-processed in MATLAB. The raw ADC codes were converted into angular-rate units using the nominal mapping between counts and degrees per second, and the resulting time series were inspected for the following:

- Bias level at zero rate (mean output in the absence of rotation);
- Short-term noise behavior;
- The absence of spurious jumps or missing samples.

These static tests confirmed that the card produces a stable output around the expected mid-scale value corresponding to zero angular rate and that the interrupt-driven acquisition and nvSRAM logging operate correctly over extended periods without data corruption.

#### 5.1.2. Real In-Lab High-Spin Test

To validate the performance of the complete DAS under realistic high-dynamic conditions, a dedicated laboratory experiment was conducted using the final integrated hardware assembly. The system, including the analog gyroscope, processing electronics, memory subsystem, power circuitry, and battery, was enclosed within a compact metallic enclosure equipped with an external ON/OFF switch. The enclosure was then securely mounted on a high-speed rotation machine capable of providing both high rotational rates and independent reference feedback measurements.

The test scenario was designed to evaluate system performance beyond the operating range of conventional MEMS-based IMUs. The rotation machine was initially accelerated to angular rates exceeding 2000°/s (2392°/s), thereby entering a region where many commercial gyro-based IMUs would experience saturation. Once the target high-spin condition was reached, the rotational speed was gradually reduced in a controlled manner until rates below 2000°/s (1960°/s) were achieved. Then, the rate was increased again to ensure that the proposed DAS is still capable of measuring correct angular rates below and above the known threshold of 2000°/s. Throughout the entire acceleration and deceleration phases, the DAS continuously acquired, processed, and logged gyroscope measurements to the onboard non-volatile memory. After finishing the aforementioned dynamic validation test, the stored data were extracted and analyzed. The time history of the angular-rate measurements showed continuous, coherent logging from the start of the experiment until the end of the recording window, with no evidence of memory corruption or loss of samples.

Following the experiment, the recorded data were extracted and processed using the calibration coefficients obtained from the laboratory turntable calibration procedure. The calibrated gyroscope measurements were then compared against the reference angular-rate feedback provided by the rotation machine. This comparison enabled evaluation of the complete sensing chain, including the gyroscope, analog signal-conditioning circuitry, analog-to-digital conversion stage, embedded firmware, memory logging subsystem, and calibration process.

**Figure 18:**
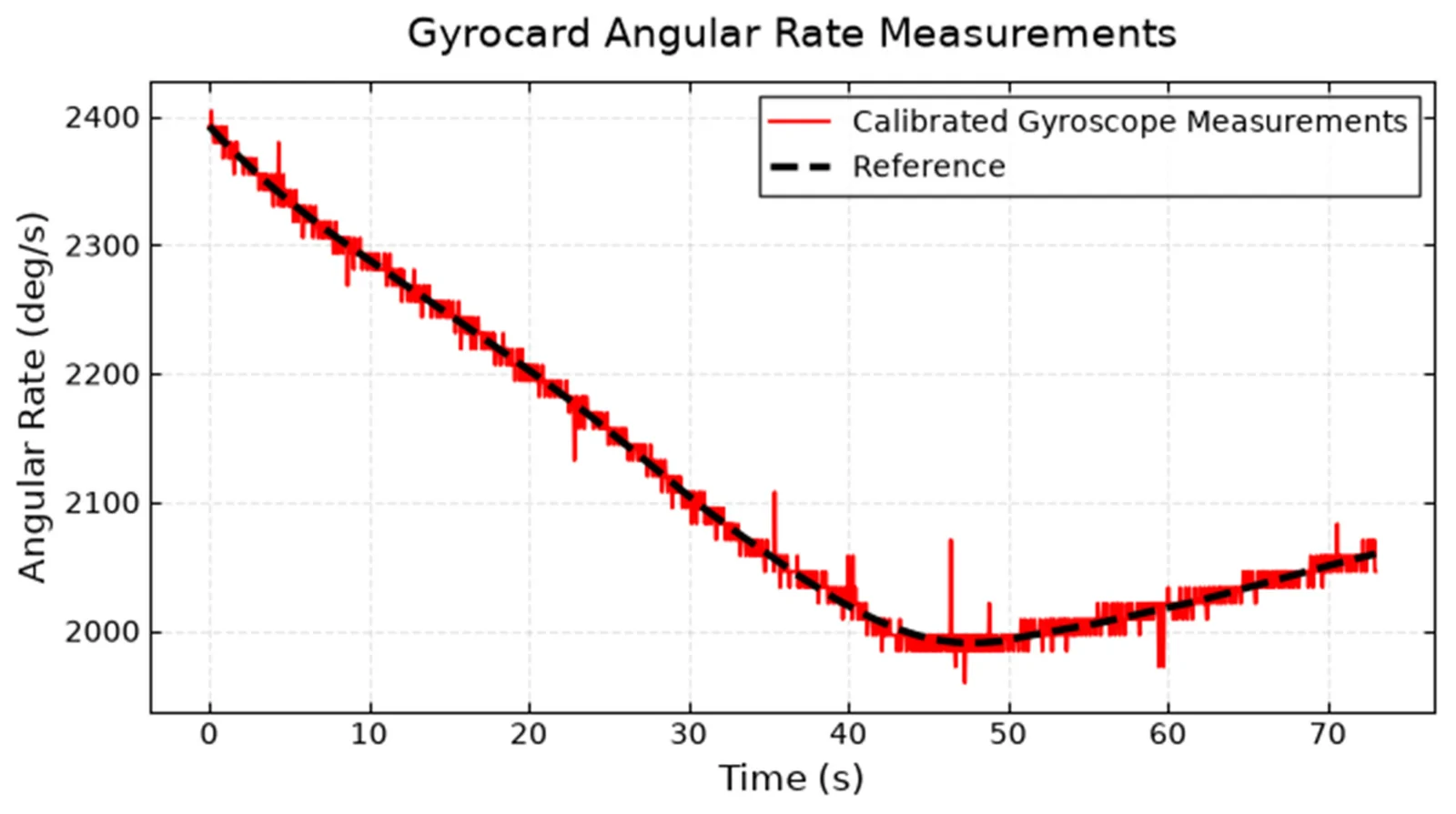
Calibrated angular rate output data from proposed analog-based DAS rotating on a turntable with spinning rate > 2000 deg/s.

The experimental results demonstrated stable operation throughout the test, with no data corruption, memory failures, or unexpected system resets observed during high-speed rotation. Furthermore, the calibrated angular-rate measurements showed good agreement with reference feedback values across both the high-spin region above 2000°/s and the lower-rate deceleration phase (see Figure 18). These results confirm the capability of the proposed system to provide reliable angular-rate measurements in extreme rotational environments while maintaining data integrity and measurement consistency.

It should be noted that the experimental calibration and validation ranges were constrained by the capabilities of the available laboratory equipment rather than by the proposed DAS or the selected ADXRS649 analog gyroscope. The deterministic calibration was performed using a laboratory turntable with a maximum angular rate of ±1000°/s, while dynamic validation was conducted using a high-speed rotary machine capable of approximately 2400°/s. These ranges were sufficient to verify the functionality of the complete sensing chain and to demonstrate reliable operation beyond the ±2000°/s limit of conventional MEMS gyro-based IMUs. Nevertheless, experimental characterization over the full ±20,000°/s measurement range remains an important direction for future work and will require access to specialized high-speed rotational test facilities. It is also important to emphasize that the objective of this work is not to develop a new calibration algorithm but rather to realize and experimentally validate a complete embedded data acquisition platform capable of supporting high-range analog gyroscopes for high-spin applications.

## 6. Conclusions and Future Work

This paper presented the design, implementation, calibration, and validation of a complete analog gyro-based Data Acquisition System (DAS) intended for high-spinning rotational-rate applications. The proposed system addresses the limitations of conventional gyro-based IMUs, whose measurement ranges are typically restricted to approximately ±2000°/s, making them unsuitable for many ultra-high-dynamic platforms. A complete hardware architecture was developed and implemented, including the selection and integration of the ADXRS649 analog gyroscope, dsPIC33 microcontroller, high-speed nvSRAM memory, power management circuitry, and signal-conditioning stages. Particular emphasis was placed on developing a compact and robust multilayer PCB capable of reliable operation under demanding environmental conditions. The resulting hardware platform provides a low-cost and scalable solution for high-rate angular measurement and data logging applications.

In addition to the hardware implementation, a comprehensive firmware architecture was developed and documented. The software design included system initialization procedures, memory integrity verification, gyroscope self-test execution, synchronized data acquisition, onboard processing, and non-volatile data storage. These features enhance system reliability and enable autonomous operation in field environments.

A detailed calibration methodology was established using a laboratory-grade turntable. Multiple clockwise (CW) and counter-clockwise (CCW) rotational datasets covering the operating range of the sensor were collected and analyzed to estimate the deterministic error parameters of the gyroscope, including bias and scale factor. The resulting calibration coefficients were subsequently applied to improve measurement accuracy and reduce systematic errors.

To validate the complete sensing chain, a real high-spin laboratory experiment was conducted using the final integrated system housed within a metallic enclosure equipped with an external ON/OFF switch. The assembled unit was mounted on a high-speed rotation machine capable of generating rotational rates exceeding 2000°/s while providing independent reference feedback measurements. Comparison between the calibrated gyroscope outputs and the machine reference data demonstrated accurate angular-rate estimation, stable operation, and reliable data integrity under extreme rotational conditions.

The obtained results confirm that analog gyroscopes, when combined with optimized acquisition electronics, robust firmware, and proper calibration procedures, remain a viable and cost-effective solution for ultra-high-spin applications. Future work will focus on extending the experimental validation of the proposed system to rotational rates approaching the full ±20,000°/s operating range of the ADXRS649 using specialized high-speed spin facilities. Additional investigations will include comprehensive temperature characterization and compensation to further improve measurement accuracy under varying environmental conditions, as well as the implementation of advanced calibration techniques incorporating stochastic error models. The use of higher-resolution data acquisition hardware and higher sampling rates while replacing the memory chip with a higher-logging-capacity one will also be explored to further enhance measurement fidelity correlated to higher data logging time for long-term missions. Finally, the proposed architecture may be extended to multi-axis inertial sensing by integrating additional gyroscopes and accelerometers while preserving the compact, ruggedized design and autonomous data-logging capability demonstrated in this work.

## Figures and Tables

**Table 1:** Comparison of representative commercial gyroscope-based IMUs and their maximum angular-rate measurement ranges.

Device	Manufacturer	Gyroscope Range (°/s)	Suitable Above 2000°/s?
MPU-6050	TDK InvenSense	±250, ±500, ±1000, ±2000	No
ICM-42688-P	TDK InvenSense	±15.6 to ±2000	No
BMI088	Bosch (Reutlingen, Germany)	±125 to ±2000	No
LSM6DSOX	STMicroelectronics (Geneva, Switzerland)	±125 to ±2000	No
ADIS16470	Analog Devices	±2000	No

**Table 2:** Comparison of ADXRS649-based angular rate DAS reported in the literature and the proposed system.

Reference	Gyros	Sampling/ADC	Storage	Health Monitoring	Experimentally Tested Angular Rate
Mi et al. [11]	3 × ADXRS649 (placed parallel on the same axis)	External acquisition	External (Not reported)	Not reported	Laboratory high-spin validation reported below ±10,000°/s
Sun et al. [16]	ADXRS649	AD7988-1 16-bit	AT25256	Motion interrupt only	No laboratory high-spin validation reported
Liu et al. [28]	ADXRS649	FPGA attached ADC 16-bit	External (Not reported)	Not reported	Rotational experiment below 2000°/s
Fuss et al. [34]	3 × ADXRS649	8-bit RISC single-chip microcontroller	Wireless transmission to smartphone/tablet	Not reported	Cricket ball spin below ±20,000°/s
Ge et al. [35]	ADXRS649	Not reported	Not reported	Not reported	Rotational turntable experiment of 60 and 90°/s
Proposed work	ADXRS649	12-bit dsPIC ADC	On-board nvSRAM	Gyroscope + memory self-test	Laboratory calibration and real high-spin test above 2000°/s

**Table 3:** Uncalibrated vs calibrated gyro measurements for multiple datasets.

Test ID	Reference Data (deg/s)	Uncalibrated Data (deg/s)	Calibrated Data (deg/s)	Error (Reference—Calibrated) (deg/s)
1	−980	−878.491339358669	−979.22344192792	−0.77655807207747
2	−960	−859.115224981864	−957.15377370234	−2.84622629765784
3	−940	−843.547712238138	−939.42215658499	−0.57784341500962
4	−920	−824.624120407671	−917.86791793770	−2.13208206229308
5	−900	−810.026208905122	−901.24069034282	1.24069034282320
6	−650	−588.715577301381	−649.16475193201	−0.83524806798209
7	−400	−369.081683517363	−398.99864160453	−1.00135839546061
8	−150	−151.373104539428	−151.02549206237	1.02549206237359
9	−65	−75.7945571704367	−64.940458037094	−0.05954196290532
10	−45	−55.6315899811474	−41.974553333127	−3.02544666687269
11	−25	−41.0132895438833	−25.324102453332	0.324102453332536
12	−5	−21.6241600696087	−3.2396098477195	−1.76039015228045
13	0	−34.8301378043149	−18.281405201088	18.2814052010884
14	5	−11.7069215400178	8.05626505297891	−3.05626505297891
15	25	1.33771564702935	22.9142912414406	2.08570875855940
16	45	21.5162862154566	45.8979684148556	−0.89796841485560
17	65	37.4555111938337	64.0529710229518	0.947028977048163
18	150	112.020579021084	148.983637521044	1.01636247895635
19	400	334.225556007710	402.078247967712	−2.07824796771246
20	650	553.044729372099	651.316380218257	−1.31638021825745
21	900	770.367743204831	898.850365608702	1.14963439129815
22	920	790.922509849824	922.262535432196	−2.26253543219582
23	940	808.019242694720	941.735955865754	−1.73595586575436
24	960	824.823938572262	960.876742064514	−0.87674206451436
25	980	842.343380883441	980.831634556673	−0.83163455667249

**Table 4:** Statistical performance of the proposed deterministic calibration.

Metric	Unit	All Test Points	Dynamic Test Points (Excluding 0°/s)
Mean Error (ME)	deg/s	≈0.000	−0.762
Mean Absolute Error (MAR)	deg/s	2.086	1.411
Root Mean Square Error (RMSE)	deg/s	3.989	1.628
Standard Deviation (Sd)	deg/s	3.989	1.439
Variance (Var)	(deg/s)^2^	15.914	2.071
Maximum Absolute Error	deg/s	18.281	3.056

## Data Availability

The original contributions presented in the study are included in the article; further inquiries can be directed to the author.
